# Application of the Variational Method to the Large Deformation Problem of Thin Cylindrical Shells with Different Moduli in Tension and Compression

**DOI:** 10.3390/ma16041686

**Published:** 2023-02-17

**Authors:** Xiao-Ting He, Xiao-Guang Wang, Jun-Yi Sun

**Affiliations:** 1 School of Civil Engineering, Chongqing University, Chongqing 400045, China; 2 Key Laboratory of New Technology for Construction of Cities in Mountain Area (Chongqing University), Ministry of Education, Chongqing 400045, China

**Keywords:** variational method, thin cylindrical shell, large deformation, tension and compression, bimodular effect

## Abstract

In this study, the variational method concerning displacement components is applied to solve the large deformation problem of a thin cylindrical shell with its four sides fully fixed and under uniformly distributed loads, in which the material that constitutes the shell has a bimodular effect, in comparison to traditional materials, that is, the material will present different moduli of elasticity when it is in tension and compression. For the purpose of the use of the displacement variational method, the physical equations on the bimodular material model and the geometrical equation under large deformation are derived first. Thereafter, the total strain potential energy is expressed in terms of the displacement component, thus bringing the possibilities for the classical Ritz method. Finally, the relationship between load and central deflection is obtained, which is validated with the numerical simulation, and the jumping phenomenon of thin cylindrical shell with a bimodular effect is analyzed. The results indicate that the bimodular effect will change the stiffness of the shell, thus resulting in the corresponding change in the deformation magnitude. When the shell is relatively thin, the bimodular effect will influence the occurrence of the jumping phenomenon of the cylindrical shell.

## 1. Introduction

Thin shell structures are favored by structural engineers because of their beautiful shape and material saving. Thin shells can make full use of the strength of materials and combine the bearing and enclosure of the structure into one; in addition, under the external loads, the shell can uniformly distribute the pressure to the various parts of the shell body, making the stress state more reasonable. These advantages enable structural engineers to extensively apply thin shells in structural design [1]. At the same time, because the shell is thin, the large deformation problem often occurs; meanwhile, the stress state of thin shells is mainly compressive, prone to the stability problem. This makes the mechanical analysis of thin shells one of the topics of interest to engineers [2]. On the other hand, with the development of materials technology, thin shells composed of advanced materials are also widely used in aircraft manufacturing and ship engineering. Due to the richness and diversity of materials, the thin shell structures can serve in more demanding environments. Therefore, the research of composite thin shells has also become a hot issue in recent decades [3,4].

Still aiming for the thin shell, in this study, a new research work considering different tensile-compressive moduli (bimodular effect) of materials [5] will be conducted. Specifically, the large deformation problem of a thin cylindrical shell with a bimodular effect is proposed first and solved via the variational method. In fact, the bimodular effect of materials exists in almost any material, regardless of whether single or composite, but obvious or not [6,7,8]. In most of the existing studies, however, it is generally assumed that the tensile modulus is the same as the compressive one, and this inevitably simplifies the theoretical analysis. Since the bimodular effect will increase the difficulty of analysis, it is not generally considered in the analysis. However, the bimodular effect may exploit the potential of materials in mechanical properties, thus providing a theoretical benchmark for the refined analysis and optimized design of structures. From this perspective, it is worthwhile to consider the bimodular effect, especially in the analysis and design of thin shells. At present, a large number of studies on shells are based on a single modulus situation, with scant literature considering the bimodular effect of materials, and most of them focus on beam and plate members. This paper aims at providing an analytical study for the large deformation problem of a thin cylindrical shell with a bimodular effect and the resulting jumping phenomenon. The whole review will begin with the model of bimodular materials and its application in the analysis of structures, followed by the concise review of thin cylindrical shells, and lastly the corresponding analytical methods aiming at the large deformation problem of plates and shells.

## 2. Background

A material that has different tensile-compressive moduli is referred to as a bimodular material [9]. Most materials—for example, ceramics, concrete, rubber, graphite and some biomedical materials—exhibit different tensile-compressive strains when they are subjected to tensile-compressive stresses of the same magnitude. Generally, there are two models widely adopted in the theoretical analysis within engineering professions. The first model is proposed by Bert [10], which is established based on the criterion of positive–negative signs in the longitudinal strain of fibers. In the analysis of orthotropic materials and laminated composites [11,12,13], the Bert model has been extensively used. The second model is the Ambartsumyan model [5] established based on the criterion of positive–negative signs of principal stresses, which is applicable to isotropic materials. The Ambartsumyan model is of particular significance in structural analysis, because it is this criterion that dictates whether a certain point of structure is tensile or compressive. The present work is carried out on the Ambartsumyan model. Because the principal stress state of a point is generally obtained as a final result but not as a known condition before solving, there is no way to use the Ambartsumyan model established based on the criterion of principal stress. Analytical solutions are available in a few simple cases, although they only concern single components—for example, beams and plates [14,15,16]. In complex cases, the finite element method (FEM) on an iterative technique has to be resorted to. In each iteration, the first step is to judge the principal stress state of each element, thus obtaining a new elastic matrix used for the next iteration. This method is called the direct iterative method with variable stiffness, which was widely adopted in some earlier studies, as indicated in the reviews of Ye et al. [17] and Sun et al. [18]. Given that the traditional iteration method struggled with the convergence difficulty, Du et al. [19] successfully established a new computational framework for these kinds of constitutive models. Thereafter, based on an improved constitutive model and combined with the arc-length method, Ma et al. [20] established a finite element iterative program for bimodular rods to obtain buckling critical loads.

Among all shell structures, cylindrical shells are a special type of shells, which is characterized by zero curvature along the longitudinal direction (the bus direction of cylinder surface); this brings convenience in the analysis, design, fabrication and construction of shell structures. Thus, cylindrical shells have been regarded as an effective structural element, which is widely used in roof construction, pressure vessels and other engineering structures. There are many review works in this field, and they involve various aspects of the analysis and application of cylindrical shells, such as the earlier reviews on the buckling of moderately thick laminated cylindrical shells [21] and on the development of layered vessels using flat-ribbon-wound cylindrical shells [22], and recent reviews on the analysis of graphene nanoplatelet-reinforced cylindrical shells subjected to thermo-mechanical loads [23], on the recent progress in lightweight carbon fiber-reinforced lattice cylindrical shells [24], on the ring stiffened cylindrical shell structures [25] and on the knockdown factor of buckling load for axially compressive cylindrical shells [26], to list but a few. More recently, the natural frequencies optimization of circular cylindrical shells using axially functionally graded materials was investigated [27]. For a prestressed cylindrical shell with various structural parameters, the experimental work of the stress state was presented [28]. For sandwich shells with functionally graded coatings while operating under different external pressures, its buckling behavior was generally investigated under simply supported boundary conditions [29]. In the existing analyses of cylindrical shells, however, few works have been found on the large deformation problems of cylindrical shells, especially on the bimodular effect of materials mentioned in this study.

In general, the analytical methods of plate and shell problems fall into the following three categories: the first is the series expansion method using different function forms (for example, power function and trigonometric function); second, variational methods established based on energy principle (for example, the Ritz method and the Galerkin method); and the third is the perturbation technique. These methods have their own advantages and disadvantages which are not the focus in this paper. As solutions to the large deformation problems of plate and shell, both the variational method and the perturbation method show their unique advantages. They have the ability to provide a more consistent solution, although the approaches obtaining the same solution are quite different. In the perturbation method, the governing equation containing the unknown functions (for example, displacement and stress) should be established first. The undetermined functions are then spread in the form of ascending powers to a certain small parameter. A series of equations determining the approximate solution of all levels are thus derived by substituting the expansion into the governing equations and boundary conditions, and then by equating the same order of the perturbation parameter. Considering that the perturbation parameter either appears explicitly or is introduced artificially, Chien [30] selected the central deflection of a thin plate as a perturbation parameter to derive successfully perturbation solution in 1947. Comparing with the experimental data, Chien’s solution is accurate and considered as a landmark, which has been cited in subsequent studies for a long period of time. In the variational method concerning displacement, first the displacement function containing undetermined coefficients is prescribed, which should satisfy the boundary conditions. At the same time, the functional of energy should be established, in which the total strain energy stored in the elastic body and the work performed by external loads are determined. By substituting the prescribed displacement into the functional of energy, the variation of displacement is only realized by the variation of coefficients, thus determining the unknown coefficients. In Chien’s perturbation solution of the large deflection problem of a thin circular plate, the relationship between load and central deflection gives *qa*^4^/64*D* = *w*_0_ [1 + 0.544(*w*_0_/*t*)^2^] [30], in which *q* is the uniformly distributed load, *a* is the radius of the thin circular plate, *D* is the bending stiffness, *t* is the plate thickness and *w*_0_ is the central deflection. For the same problem, the variational method gives the solution *qa*^4^/64*D* = *w*_0_ [1 + 0.486(*w*_0_/*t*)^2^] [31]. It is easy to see that they are quite close. In fact, in the previous study [32], the variational method was successfully used in the analysis of stability of cantilever vertical plates with different moduli. In addition, the recent study [33] also showed that, for the large deformation of thin shallow spherical shells, the perturbation solution agrees well with the variational solution. All these works showed that the variational method can be used to solve the large deformation problem of plate and shell.

In conclusion, the variational method has two advantages in this study. First, since the displacement variational equation itself stands for the equation of equilibrium and stress boundary conditions, it naturally avoids the equilibrium condition of shells. It is known that for cylindrical shell problems, the establishment of the equation of equilibrium is somewhat complicated. Second, the analytical characteristic of the variational method also makes it more conveniently used in the sequent parametric study and in the design of the shell. In this study, the variational method of displacement is applied to solve the large deformation problem of bimodular cylindrical shells. To this end, this paper is organized as follows. In Section 3, the variational method used and the cylindrical shell problem studied are briefly described. In Section 4 and Section 5, the physical equations on the bimodular material model and the geometrical equations under the large deformation are derived, respectively. In Section 6, the total strain potential energy will be first derived and then the Ritz method is applied to solve the large deformation of bimodular cylindrical shells, thus the relationship between load and central deflection is obtained. Section 7 contains the numerical simulation and comparison with theoretical results. In Section 8, the influences of relevant parameters on this relation as well as the bimodular effect on the jumping phenomenon are discussed in detail. The concluding remarks are summarized in Section 9.

## 3. Method and Problem

### 3.1. The Displacement Variational Method Based on Energy Conservation

In a three-dimensional spatial problem of elasticity whose orthogonal coordinate system is established as *o-xyz*, let *σ_x_*, *σ_y_*, *σ_z_*, *τ_xy_*, *τ_yz_* and *τ_zx_* be the six stress components and let *ε_x_*, *ε_y_*, *ε_z_*, *γ_xy_*, *γ_yz_* and *γ_zx_* be the six corresponding strain components, so that the strain potential energy of the whole elastic body, *U*, will be expressed in terms of stress and strain components as [31]

(1) $$U=\frac{1}{2}\int \int \int (\sigma_{x}\varepsilon_{x}+\sigma_{y}\varepsilon_{y}++\sigma_{z}\varepsilon_{z}+\tau_{xy}\gamma_{xy}+\tau_{yz}\gamma_{yz}+\tau_{zx}\gamma_{zx})dxdydz.$$

Via the geometrical equation and the physical equation of the three-dimensional problem, Equation (1) may also be expressed in terms of the displacement components [31],

(2) $$U=\frac{E}{2(1+\mu )}∭\left[\begin{array}{l} \frac{\mu }{1-2\mu }{\left(\frac{\partial u}{\partial x}+\frac{\partial v}{\partial y}+\frac{\partial w}{\partial z}\right)}^{2}+{\left(\frac{\partial u}{\partial x}\right)}^{2}+{\left(\frac{\partial v}{\partial y}\right)}^{2}+{\left(\frac{\partial w}{\partial z}\right)}^{2}\\ +\frac{1}{2}{\left(\frac{\partial v}{\partial x}+\frac{\partial u}{\partial y}\right)}^{2}+\frac{1}{2}{\left(\frac{\partial w}{\partial y}+\frac{\partial v}{\partial z}\right)}^{2}+\frac{1}{2}{\left(\frac{\partial u}{\partial z}+\frac{\partial w}{\partial x}\right)}^{2} \end{array}\right]dxdydz,$$

in which *u*, *v* and *w* are the displacement components along the *x*, *y* and *z* directions, respectively. Obviously, Equation (2) opens the possibilities for the use of the displacement variational method based on energy conservation.

Suppose that an arbitrary elastic body is under the action of the external forces including the body force along the *x*, *y* and *z* directions, *X*, *Y* and *Z*, and the surface force along the *x*, *y* and *z* directions, $\bar{X}$, $\bar{Y}$ and $\bar{Z}$, and now it is in equilibrium. The resulting displacement components, *u*, *v* and *w*, should satisfy the differential equation of equilibrium expressed in terms of displacement, displacement boundary conditions as well as stress boundary conditions expressed in terms of displacement. If the displacements take place minor changes allowed by boundary conditions, the new displacement will become

(3) $$u^{*}=u+\delta u,\;v^{*}=v+\delta v,\;w^{*}=w+\delta w,$$

where *δu*, *δv* and *δw* are the virtual displacement or displacement variation. During the virtual displacement, if there are no any changes in thermal energy and kinetic energy, according to the principle of conservation of energy, the increment of strain potential energy, *δU*, should be equal to the work done by the external forces, thus the following displacement variational equation [31] may be obtained

(4) $$\delta U=∭\left(X\delta u+Y\delta v+Z\delta w\right)dxdydz+\iint (\bar{X}\delta u+\bar{Y}\delta v+\bar{Z}\delta w)dS,$$

which also may be referred to as the Lagrangian variational equation. This variational equation provides an approximate solution to problems of elasticity. Specifically, if a group of displacement components containing a series of undetermined coefficients may satisfy the displacement boundary conditions, Equation (4) may be adopted to determine these unknown coefficients, thus obtaining the final displacement.

If the expression of the displacement component is taken as follows:

(5) $$u=u_{0}+\sum_{m}A_{m}u_{m},\;v=v_{0}+\sum_{m}B_{m}v_{m},\;w=w_{0}+\sum_{m}C_{m}w_{m},$$

in which *A_m_*, *B_m_* and *C_m_* are the independent coefficients whose number is 3*m*; *u*_0_, *v*_0_ and *w*_0_ are the specified functions whose boundary values are equal to the known displacements on the boundaries; and *u_m_*, *v_m_* and *w_m_* are specified functions which are equal to zero on the boundaries. Thus, no matter what the coefficients, *A_m_*, *B_m_* and *C_m_*, are taken, the displacement components, *u*, *v* and *w*, always satisfy the boundary conditions. Note that the displacement variation is realized only by the variation of *A_m_*, *B_m_* and *C_m_*, according to Equation (5), the variation of the displacement components are

(6) $$\delta u=\sum_{m}u_{m}\delta A_{m},\;\delta v=\sum_{m}v_{m}\delta B_{m},\;\delta w=\sum_{m}w_{m}\delta C_{m},$$

While the variation of the strain potential energy is

(7) $$\delta U=\sum_{m}\left(\frac{\partial U}{\partial A_{m}}\delta A_{m}+\frac{\partial U}{\partial B_{m}}\delta B_{m}+\frac{\partial U}{\partial C_{m}}\delta C_{m}\right).$$

Substituting Equations (6) and (7) into Equation (4), and also merging these terms with the same coefficient, Equation (4) may be changed as

(8) $$\begin{array}{l} \sum_{m}\left(\frac{\partial U}{\partial A_{m}}-∭Xu_{m}dxdydz-\iint \bar{X}u_{m}dS\right)\delta A_{m}\\ +\sum_{m}\left(\frac{\partial U}{\partial B_{m}}-∭Yv_{m}dxdydz-\iint \bar{Y}v_{m}dS\right)\delta B_{m}+\\ +\sum_{m}\left(\frac{\partial U}{\partial C_{m}}-∭Zw_{m}dxdydz-\iint \bar{Z}w_{m}dS\right)\delta C_{m}=0 \end{array}.$$

Since the variations, *δA_m_*, *δB_m_* and *δC_m_*, are completely arbitrary and independent each other, the coefficients of these variations in Equation (8) must be equal to zero, thus the following three relations may be obtained

(9) $$\left\{ \begin{array}{l} \frac{\partial U}{\partial A_{m}}=∭Xu_{m}dxdydz+\iint \bar{X}u_{m}dS\\ \frac{\partial U}{\partial B_{m}}=∭Yv_{m}dxdydz+\iint \bar{Y}v_{m}dS\\ \frac{\partial U}{\partial C_{m}}=∭Zw_{m}dxdydz+\iint \bar{Z}w_{m}dS \end{array},\right.$$

which may be used for the solution of the undermined coefficients, thus the displacement components may be finally obtained via Equation (5). In many studies [31,34,35], this displacement variational method is also referred to as the Ritz method.

### 3.2. Describtion of Problem

**Figure 1:**
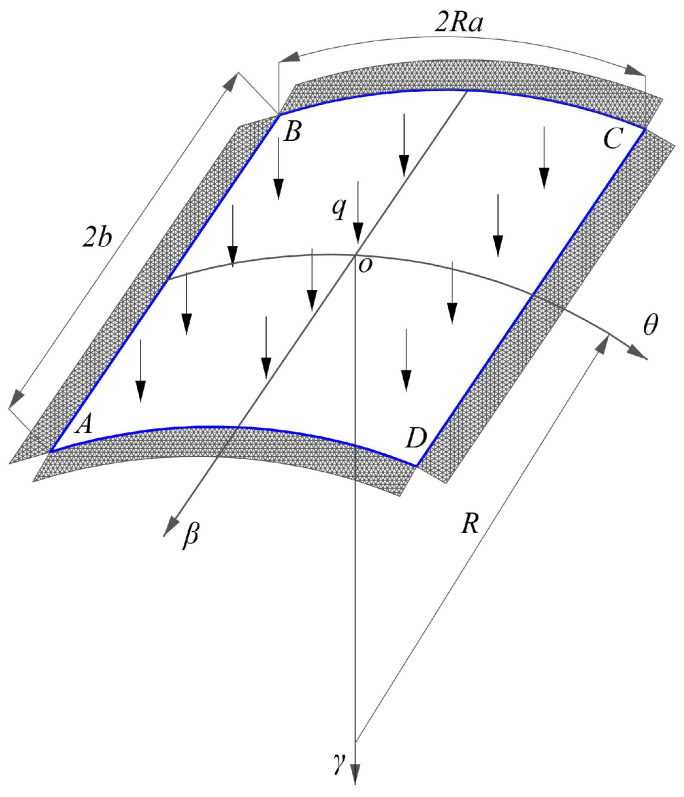
The bimodular cylindrical shell under transversely uniformly distributed load.

As shown in Figure 1, a bimodular thin cylindrical shell with its four sides fully fixed is subjected to a vertically uniformly distributed load *q*, in which the four corner points of the shell are expressed in points *A*, *B*, *C*, and *D*. The orthogonal curvilinear coordinate systems (*θ*, *β*, *γ*) is established, in which the *β* coordinate axis is placed along the longitudinal direction, that is, the bus direction of the cylinder surface; the *θ* coordinate axis (in radians) is placed along the circumferential direction, that is, the quasi-line direction of the cylinder surface; and the *γ* coordinate axis is placed on the direction normal to the curved plane (*θ*, *β*), whose positive direction is along the direction of the concave surface of the shell (downward). To facilitate the following analysis, the origin *o* is placed at the center of the shell, as shown in Figure 1. The overall size of the shell is as follows: the length of the shell along the *β* direction is 2*b*, the arc length of the shell along the *θ* direction is 2*Ra* in which *a* (in radians) is the half of the central angle of the cylindrical surface and *R* is the radius of the cylindrical surface. Note that except the load *q* whose unit is Pa, the units of *β*, *γ*, *b* and *R* are all length, while *θ* and *a* are in radians thus they are dimensionless quantities. Due to the geometrical characteristic of the cylindrical shell, the curvature along the *θ* direction is *k*_1_ = 1/*R*, while the curvature along the *β* direction is *k*_2_ = 0.

It should be noted here that the constraints for the thin cylindrical shell is considered as fully fixed on four sides. Doing so is based on the following two reasons. First, to facilitate the application of the variational method of displacement, the prescribed displacement should satisfy all displacement boundary conditions and fully fixed conditions is easily expressed in terms of the displacement components. Second, from the perspective of the application, most of the roof structures tend to be the constraints such as this, with a certain representative.

The cylindrical shell considered in this study has two distinct features, as compared to the other cylindrical shells. One is the shell will undergo the large deformation and another is the material composed the shell has a bimodular effect. From the above analysis, it is easy to see that for the application of the displacement variational method, all stress and strain components should be expressed in the form of the displacement components; thus in the next analysis, the first step is to derive the physical equation on the bimodular material model and the geometrical equation under large deformation. Due to the introduction of two features mentioned above, the physical equation and the geometrical equation will inevitably change to some extent, compared to the common equations.

## 4. Physical Equations on Bimodular Materials Model

**Figure 2:**
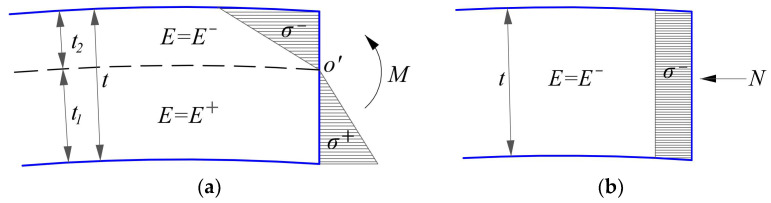
The determination of the tension-compression moduli (**a**) under pure bending and (**b**) under in-plane compressive stress.

The material considered in this work has a bimodular effect, that is, when the material is in tension or in compression, it will behave different Young’s moduli of elasticity. As shown in Figure 1, when a thin cylindrical shell is subjected to the uniformly distributed loads acting vertically downward, there will be two different responses generated to resist the external loads, one is the bending effect and another is the in-plane compression effect, as shown in Figure 2a and Figure 2b, respectively. These two effects act independently and not affect each other, so they can be considered separately. In other words, in each effect, if the stress state is known—for example, whether it is pulled or pressed—then the elastic modulus (tensile modulus or compressive one) can be selected accordingly. More specifically, when a certain section of the shell is under pure bending, as shown in Figure 2a, in which *M* is the bending moment, *o*’ is the center of bending moment, the resulting tension-compression moduli may be determined like this. Bounded by the unknown neutral layer (which is marked in dashed line), the area above the neutral layer is in compression and the stress is compressive *σ*^−^ thus the corresponding elastic modulus should be *E*^−^; while the area below the neutral layer is in tension and the stress is tensile *σ*^+^ thus the corresponding elastic modulus should be *E*^+^. Due to the tension-compression subareas under pure bending, the whole thickness of the shell, *t*, is divided into the tensile thickness and compressive one, which is denoted by *t*_1_ and *t*_2_, respectively, as shown in Figure 2a. The determination of *t*_1_ and *t*_2_ may come from the bimodular thin plate under pure bending— for example, the previous study gives [36]

(10) $$\left\{ \begin{array}{l} \frac{t_{1}}{t}=\frac{\sqrt{E^{-}[1-{\left(\mu^{+}\right)}^{2}]}}{\sqrt{E^{+}[1-{\left(\mu^{-}\right)}^{2}]}+\sqrt{E^{-}[1-{\left(\mu^{+}\right)}^{2}]}}\\ \frac{t_{2}}{t}=\frac{\sqrt{E^{+}[1-{\left(\mu^{-}\right)}^{2}]}}{\sqrt{E^{+}[1-{\left(\mu^{-}\right)}^{2}]}+\sqrt{E^{-}[1-{\left(\mu^{+}\right)}^{2}]}} \end{array},\right.$$

where *μ*^+^ and *μ*^−^ are the tensile and compressive Poisson’s ratio, respectively. In addition, Figure 2b shows the stress state of the section of the shell under in-plane loads. From the load type depicted in Figure 1, the in-plane stresses are basically compressive, thus the elastic modulus along the whole thickness may be taken as *E*^−^.

**Figure 3:**
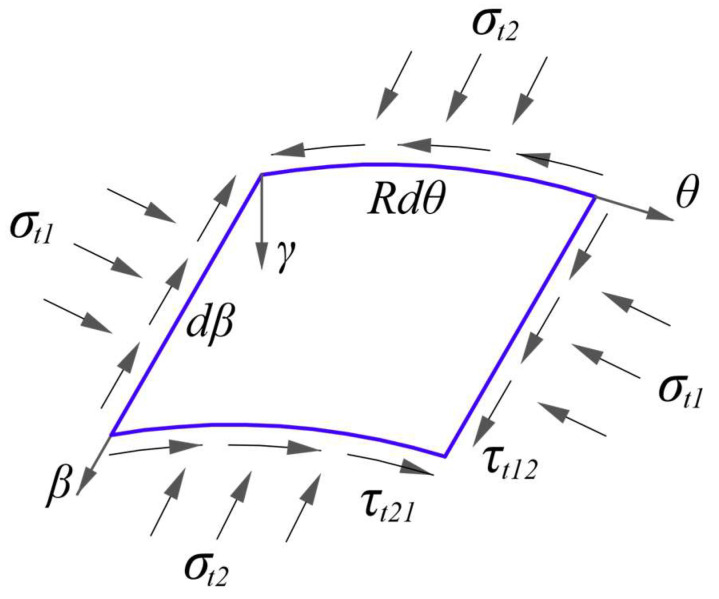
In-plane stress components.

According to the basic assumptions of the common thin cylindrical shell, the transverse shear stresses are not considered in the problem since the shell is thin and the deformation are mainly due to bending and torsion, thus ignoring the transverse shear stresses is acceptable. In addition, there are three in-plane stresses acting on the middle surface of the shell and they are the longitudinal stress, the circumferential stress and the shearing stress. If let subscript 1 denote the longitudinal, let subscript 2 denote the circumferential, let subscript 12 denote the corresponding shearing quantity between longitudinal and circumferential directions, and the subscript *t* denote the in-plane, thus the three in-plane stresses may be denoted by *σ_t_*_1_, *σ_t_*_2_ and *τ_t_*_12_, as shown in Figure 3. Note that according to the above description, under the action of external uniformly distributed loads, the in-plane stresses acting on the whole thickness of the section tend to be compressive, thus *σ_t_*_1_, *σ_t_*_2_ and *τ_t_*_12_ may be changed as $\sigma_{t1}^{-}$, $\sigma_{t2}^{-}$ and $\tau_{t12}^{-}$ and also the corresponding modulus of elasticity and Poisson’s ratio may be selected as *E*^−^ and *μ*^−^. The physical equation concerning the in-plane deformation may be expressed as [31]

(11) $$\left\{ \begin{array}{l} \sigma_{t1}^{-}=\frac{E^{-}}{1-{\left(\mu^{-}\right)}^{2}}(\varepsilon_{t1}^{}+\mu^{-}\varepsilon_{t2}^{})\\ \sigma_{t2}^{-}=\frac{E^{-}}{1-{\left(\mu^{-}\right)}^{2}}(\varepsilon_{t2}^{}+\mu^{-}\varepsilon_{t1}^{})\\ \tau_{t12}^{-}=\frac{E^{-}}{2(1+\mu^{-})}\varepsilon_{t12}^{} \end{array}\right.,\;\mathrm{at}\;-t_{2}\leq \gamma \leq t_{1},$$

where *ε_t_*_1_, *ε_t_*_2_ and *ε_t_*_12_ denote the corresponding strains.

On the other hand, the bending and torsion deformation of the cylindrical shell must be considered under large deformation. According to the above analysis, the physical equations concerning bending stress and torsional stress will give as follows, in subareas of tension and compression,

(12) $$\left\{ \begin{array}{l} \sigma_{m1}^{+}=\frac{E^{+}}{1-{\left(\mu^{+}\right)}^{2}}(\varepsilon_{m1}^{}+\mu^{+}\varepsilon_{m2}^{})\\ \sigma_{m2}^{+}=\frac{E^{+}}{1-{\left(\mu^{+}\right)}^{2}}(\varepsilon_{m2}^{}+\mu^{+}\varepsilon_{m1}^{})\\ \tau_{m12}^{+}=\frac{E^{+}}{2(1+\mu^{+})}\varepsilon_{m12}^{} \end{array}\right.\;\mathrm{at}\;0\leq \gamma \leq t_{1},$$

and

(13) $$\left\{ \begin{array}{l} \sigma_{m1}^{-}=\frac{E^{-}}{1-{\left(\mu^{-}\right)}^{2}}(\varepsilon_{m1}^{}+\mu^{-}\varepsilon_{m2}^{})\\ \sigma_{m2}^{-}=\frac{E^{-}}{1-{\left(\mu^{-}\right)}^{2}}(\varepsilon_{m2}^{}+\mu^{-}\varepsilon_{m1}^{})\\ \tau_{m12}^{-}=\frac{E^{-}}{2(1+\mu^{-})}\varepsilon_{m12}^{} \end{array}\right.\;\mathrm{at}\;-t_{2}\leq \gamma \leq 0,$$

where the subscript *m* denotes the out-of-plane bending or torsion; to differentiate from the subscript *t* (denote the in-plane), the superscript + denote the tensile and superscript − denote the compressive. σ*_m_*_1_ and σ*_m_*_2_ are the bending stresses, their resultants being the bending moments; *τ_m_*_12_ is the shear stress, its resultant being the torsion moment.

## 5. Geometrical Equations under Large Deformation

Generally speaking, the geometrical relations of thin cylindrical shells include two parts: one is the geometrical relation concerning bending and torsion moments, which is characterized by the second derivative of the deflection *w*; another involves the geometrical relation concerning in-plane deformation, not only relating to the *u* and *v*, but also to the *w*. However, most of the existing studies deal only with the small deformation. Under large deformation, the first geometrical relation mentioned above keeps unchanged while the second geometrical relation changes greatly, thus the geometrical equation under the large deformation is relatively complicated.

### 5.1. Geometrical Equation Concerning Bending and Torsion Moments

The geometrical relation concerning bending and torsion moments may be expressed in terms of the curvature due to bending (double curvature with same sign) and the curvature due to torsion (anticlastic—double curvature with opposite sign) [31] as follows,

(14) $$\left\{ \begin{array}{l} \varepsilon_{m1}=-\frac{1}{R^{2}}\frac{\partial^{2}w}{\partial \theta^{2}}\gamma \\ \varepsilon_{m2}=-\frac{\partial^{2}w}{\partial \beta^{2}}\gamma \\ \varepsilon_{m12}=-2\frac{1}{R}\frac{\partial^{2}w}{\partial \theta \partial \beta }\gamma \end{array},\right.$$

where *ε_m_*_1_, *ε_m_*_2_ and *ε_m_*_12_ are the strains, *w* is the deflection and *R* is the radius of the cylindrical surface. Equation (14) may be used for the computation of the strain energies in bending and torsion.

### 5.2. Geometrical Equation Concerning In-Plane Deformation

Under large deformation, the strain of the middle surface generally involves the following three aspects of deformation: the first is the strain due to the displacements *u* and *v* along the directions *θ* and *β*, which is denoted by ε*; the second is the strain due to the change in the curvature radius of shell introduced by *w*, denoted by ε^**^; and the third one is the strain ε^***^ also introduced by *w*, but only under large deformation. That is, the final strain *ε_t_* will be the sum of the three strains,

(15) $$\varepsilon_{t}=\varepsilon^{*}+\varepsilon^{**}+\varepsilon^{***}.$$

First, the middle surface strain due to the displacements *u* and *v* give [31]

(16) $$\left\{ \begin{array}{l} \varepsilon_{1}^{*}=\frac{1}{R}\frac{\partial u}{\partial \theta }\\ \varepsilon_{2}^{*}=\frac{\partial v}{\partial \beta }\\ \varepsilon_{12}^{*}=\frac{\partial u}{\partial \beta }+\frac{1}{R}\frac{\partial v}{\partial \theta } \end{array}.\right.$$

**Figure 4:**
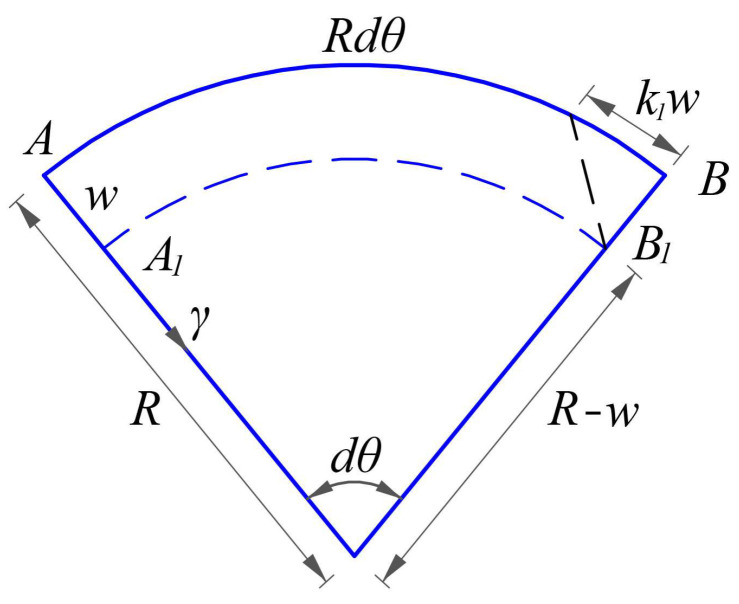
The strain *ε*_1_^**^ due to the change in the curvature radius of shell introduced by *w*.

The strain *ε*_1_^**^ (along the *θ* direction) due to the change in the curvature radius of shell introduced by *w* may refer to Figure 4, in which the arc segment *AB* along the *θ* direction is isolated from the shell body and moves to the arc segment *A*_1_*B*_1_, after taking place a positive displacement *w*. The curvature radius of the shell is denoted by *R*, thus the initial curvature of the arc segment *AB* before deformation is *k*_1_ = 1/*R*. The new curvature radius after deformation is *R*−*w*, and *dθ* is the central angle of the arc segment *AB*, thus the normal strain *ε*_1_^**^ along the *θ* direction introduced by the positive deflection *w*, will give

(17) $$\varepsilon_{1}^{**}=\frac{(R-w)d\theta -Rd\theta }{Rd\theta }=-\frac{w}{R}=-k_{1}w.$$

Due to the geometrical characteristic of the cylindrical shell, the curvature *k*_2_ along the *β* direction is zero, resulting in the other strain introduced by *w*, *ε*_2_^**^, is zero. In addition, the shear strain, *ε*_12_^**^, represents the change in the right angle between the *θ* and *β* directions. Now, the strain along the *θ* direction gives −*k*_1_*w*, which always takes place in the plane normal to the *β* direction while the strain along the *β* direction is always zero, thus the right angle between the *θ* and *β* directions cannot change, the shear strain, *ε*_12_^**^, should be zero. Thus,

(18) $$\left\{ \begin{array}{l} \varepsilon_{1}^{**}=-k_{1}w\\ \varepsilon_{2}^{**}=0\\ \varepsilon_{12}^{**}=0 \end{array}.\right.$$

**Figure 5:**
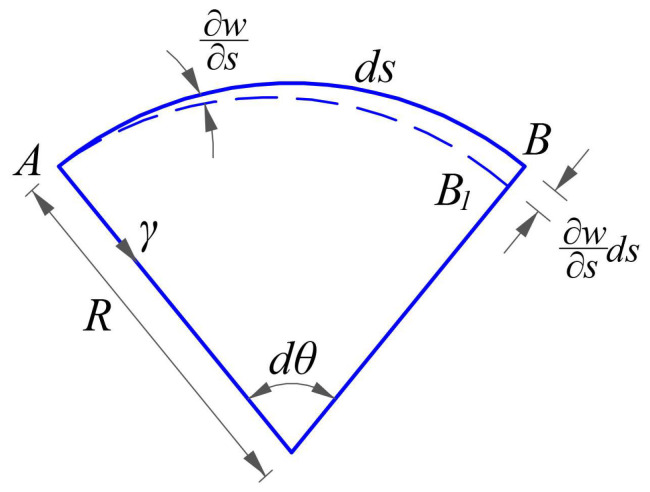
The strain *ε*_1_^***^ due to slope change in the segment along the *θ* direction introduced by *w*.

The strain *ε*_1_^***^ (along the *θ* direction) introduced by *w* may refer to Figure 5, in which the arc segment *AB* along the *θ* direction whose arc length is *ds*, now changes to *AB*_1_ after taking place a rotation change ∂w/∂s. Thus, the normal strain along the *θ* direction may be computed as

(19) $$\varepsilon_{1}^{***}=\frac{AB_{1}-AB}{AB}=\frac{\sqrt{{\left(ds\right)}^{2}+{\left(\frac{\partial w}{\partial s}ds\right)}^{2}}-ds}{ds}.$$

Spreading the expression in the square root by Taylor series, that is,

(20) $$\varepsilon_{1}^{***}=\frac{ds\left[1+\frac{1}{2}{\left(\frac{\partial w}{\partial s}\right)}^{2}+\cdot \cdot \cdot \right]-ds}{ds}\approx \frac{1}{2}{\left(\frac{\partial w}{\partial s}\right)}^{2}.$$

Note that

(21) $$\frac{\partial w}{\partial s}=\frac{1}{R}\frac{\partial w}{\partial \theta },$$

Equation (20) may be written as

(22) $$\varepsilon_{1}^{***}=\frac{1}{2R^{2}}{\left(\frac{\partial w}{\partial \theta }\right)}^{2}.$$

Similarly, *ε*_2_^***^ due to the slope change in the segment along the *β* direction introduce by *w*, may be obtained as follows

(23) $$\varepsilon_{2}^{***}=\frac{1}{2}{\left(\frac{\partial w}{\partial \beta }\right)}^{2}.$$

**Figure 6:**
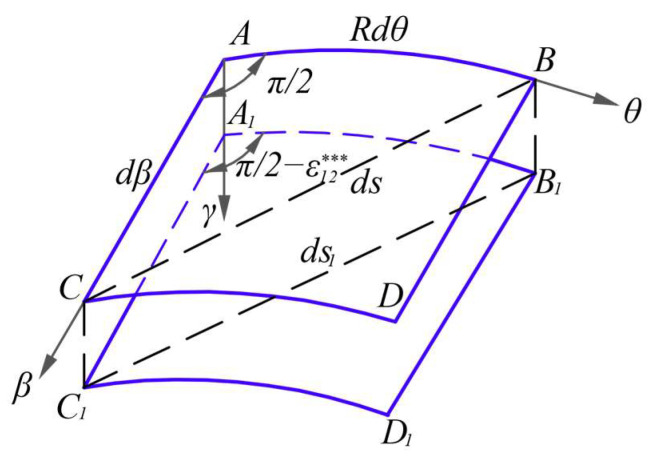
The shearing strain *ε*_12_^***^ introduced by *w*.

The shearing strain *ε*_12_^***^ introduced by *w* may refer to Figure 6, in which the differential element *ABDC* changes to *A*_1_*B*_1_*D*_1_*C*_1_, after taking place the deflection *w*. The initial included angle between *AB* and *AC* is π/2, and after taking place the shear strain *ε*_12_^***^, the included angle between *A*_1_*B*_1_ and *A*_1_*C*_1_ is π/2−*ε*_12_^***^. Additionally, let the distance between points *B* and *C* be *ds* while the distance between points *B*_1_ and *C*_1_ be *ds*_1_. Thus, according to Figure 6, there is the following geometrical relation

(24) $${\left(ds_{1}\right)}^{2}={\left(ds\right)}^{2}+{\left(\bar{CC_{1}}-\bar{BB_{1}}\right)}^{2}={\left(Rd\theta \right)}^{2}+{\left(d\beta \right)}^{2}+{\left(\frac{\partial w}{\partial \beta }d\beta -\frac{\partial w}{\partial \theta }d\theta \right)}^{2}.$$

In the triangle *A*_1_*B*_1_*C*_1_, the length of *ds*_1_ may be computed as follows, via cosine theorem,

(25) $${\left(ds_{1}\right)}^{2}={\left(\bar{A_{1}B_{1}}\right)}^{2}+{\left(\bar{A_{1}C_{1}}\right)}^{2}-2(\bar{A_{1}B_{1}})(\bar{A_{1}C_{1}})\cos\left(\frac{\pi }{2}-\varepsilon_{12}^{***}\right)$$

where

(26) $${\left(\bar{A_{1}B_{1}}\right)}^{2}={\left(Rd\theta \right)}^{2}+{\left(\frac{1}{R}\frac{\partial w}{\partial \theta }Rd\theta \right)}^{2}={\left(Rd\theta \right)}^{2}[1+{\left(\frac{1}{R}\frac{\partial w}{\partial \theta }\right)}^{2}],$$

and similarly,

(27) $${\left(\bar{A_{1}C_{1}}\right)}^{2}={\left(d\beta \right)}^{2}\left[1+{\left(\frac{\partial w}{\partial \beta }\right)}^{2}\right].$$

Combining Equations (24) and (25) yields

(28) $$\sqrt{1+{\left(\frac{1}{R}\frac{\partial w}{\partial \theta }\right)}^{2}}\sqrt{1+{\left(\frac{\partial w}{\partial \beta }\right)}^{2}}\cos\left(\frac{\pi }{2}-\varepsilon_{12}^{***}\right)=\frac{1}{R}\frac{\partial w}{\partial \beta }\frac{\partial w}{\partial \theta }.$$

Due to

(29) $${\left(\frac{1}{R}\frac{\partial w}{\partial \theta }\right)}^{2}\ll 1,{\left(\frac{\partial w}{\partial \beta }\right)}^{2}\ll 1,\cos\left(\frac{\pi }{2}-\varepsilon_{12}^{***}\right)=\sin\varepsilon_{12}^{***}\approx \varepsilon_{12}^{***},$$

the following strain will be obtained

(30) $$\varepsilon_{12}^{***}=\frac{1}{R}\frac{\partial w}{\partial \beta }\frac{\partial w}{\partial \theta }.$$

Thus, collecting the strain only under large deformation introduced by *w* will yield

(31) $$\left\{ \begin{array}{l} \varepsilon_{1}^{***}=\frac{1}{2R^{2}}{\left(\frac{\partial w}{\partial \theta }\right)}^{2}\\ \varepsilon_{2}^{***}=\frac{1}{2}{\left(\frac{\partial w}{\partial \beta }\right)}^{2}\\ \varepsilon_{12}^{***}=\frac{1}{R}\frac{\partial w}{\partial \beta }\frac{\partial w}{\partial \theta } \end{array}\right..$$

By adding Equations (16), (18) and (31), the geometrical equation concerning middle surface deformation is finally obtained as follows

(32) $$\left\{ \begin{array}{l} \varepsilon_{t1}^{}=\frac{1}{R}\frac{\partial u}{\partial \theta }-\frac{w}{R}+\frac{1}{2R^{2}}{\left(\frac{\partial w}{\partial \theta }\right)}^{2}\\ \varepsilon_{t2}^{}=\frac{\partial v}{\partial \beta }+\frac{1}{2}{\left(\frac{\partial w}{\partial \beta }\right)}^{2}\\ \varepsilon_{t12}^{}=\frac{\partial u}{\partial \beta }+\frac{1}{R}\frac{\partial v}{\partial \theta }+\frac{1}{R}\frac{\partial w}{\partial \beta }\frac{\partial w}{\partial \theta } \end{array}\right..$$

## 6. Application of the Displacement Variational Method

### 6.1. Total Strain Potencial Energy

The total strain potential energy, *U*, consists of the strain energy due to the deformation of middle surface, *U_t_*, and the energy due to the deformation of bending and torsion, *U_m_*, that is [31],

(33) $$U=U_{t}+U_{m},$$

where the subscript *t* denotes the in-plane and the subscript *m* denotes the bending or torsion, corresponding to the notational conventions in the geometrical and physical equations above.

First, the strain energy due to the deformation of the middle surface, *U_t_*, is computed as follows [31]

(34) $$U_{t}=\frac{1}{2}\underset{V}{∭}\left(\sigma_{t1}^{-}\varepsilon_{t1}+\sigma_{t2}^{-}\varepsilon_{t2}+\tau_{t12}^{-}\varepsilon_{t12}\right)dV.$$

Substituting Equation (11) into Equation (34) and also noting that the lower limit to upper limit of integration along the *γ* direction gives –*t*_2_ to *t*_1_, Equation (34) may be written as

(35) $$U_{t}=\frac{1}{2}\frac{E^{-}}{1-{\left(\mu^{-}\right)}^{2}}\int_{-t_{2}}^{t_{1}}d\gamma \iint_{S}[{\left(\varepsilon_{t1}\right)}^{2}+{\left(\varepsilon_{t2}\right)}^{2}+2\mu^{-}\varepsilon_{t1}\varepsilon_{t2}+\frac{1}{2}(1-\mu^{-}){\left(\varepsilon_{t12}\right)}^{2}]dS.$$

Additionally, substituting Equation (32) into Equation (35) will yield

(36) $$U_{t}=\frac{1}{2}\frac{E^{-}t}{1-{\left(\mu^{-}\right)}^{2}}\iint_{S}\left\{\begin{array}{l} {\left[\frac{1}{R}\frac{\partial u}{\partial \theta }-\frac{w}{R}+\frac{1}{2R^{2}}{\left(\frac{\partial w}{\partial \theta }\right)}^{2}\right]}^{2}+{\left[\frac{\partial v}{\partial \beta }+\frac{1}{2}{\left(\frac{\partial w}{\partial \beta }\right)}^{2}\right]}^{2}\\ +2\mu^{-}\left[\frac{1}{R}\frac{\partial u}{\partial \theta }-\frac{w}{R}+\frac{1}{2R^{2}}{\left(\frac{\partial w}{\partial \theta }\right)}^{2}\right]\left[\frac{\partial v}{\partial \beta }+\frac{1}{2}{\left(\frac{\partial w}{\partial \beta }\right)}^{2}\right]\\ +\frac{1}{2}(1-\mu^{-}){\left(\frac{\partial u}{\partial \beta }+\frac{1}{R}\frac{\partial v}{\partial \theta }+\frac{1}{R}\frac{\partial w}{\partial \theta }\frac{\partial w}{\partial \beta }\right)}^{2} \end{array}\right\}dS.$$

Thus, the *U_t_* is expressed in terms of *u*, *v* and *w*.

The energy due to the deformation of bending and torsion, *U_m_*, may be derived, via the subareas in tension and compression indicated above, that is,

(37) $$U_{m}=\frac{1}{2}\underset{V^{+}}{∭}\left(\sigma_{m1}^{+}\varepsilon_{m1}+\sigma_{m2}^{+}\varepsilon_{m2}+\tau_{m12}^{+}\varepsilon_{m12}\right)dV+\frac{1}{2}\underset{V^{-}}{∭}\left(\sigma_{m1}^{-}\varepsilon_{m1}+\sigma_{m2}^{-}\varepsilon_{m2}+\tau_{m12}^{-}\varepsilon_{m12}\right)dV.$$

Substituting Equations (12)–(14) into the above equation, also noting that integration limits in the tensile term is from 0 to *t*_1_ while the limits in the compressive term is from −*t*_2_ to 0, will yield

(38) $$\begin{array}{l} U_{m}\\ =\frac{1}{2}\frac{E^{+}}{1-{\left(\mu^{+}\right)}^{2}}\int_{0}^{t_{1}}\gamma^{2}d\gamma \iint_{S}\left\{{\left(\frac{1}{R^{2}}\frac{\partial^{2}w}{\partial \theta^{2}}+\frac{\partial^{2}w}{\partial \beta^{2}}\right)}^{2}-2(1-\mu^{+})\left[\frac{1}{R^{2}}\frac{\partial^{2}w}{\partial \theta^{2}}\frac{\partial^{2}w}{\partial \beta^{2}}-\frac{1}{R^{2}}{\left(\frac{\partial^{2}w}{\partial \theta \partial \beta }\right)}^{2}\right]\right\}dS\\ +\frac{1}{2}\frac{E^{-}}{1-{\left(\mu^{-}\right)}^{2}}\int_{-t_{2}}^{0}\gamma^{2}d\gamma \iint_{S}\left\{{\left(\frac{1}{R^{2}}\frac{\partial^{2}w}{\partial \theta^{2}}+\frac{\partial^{2}w}{\partial \beta^{2}}\right)}^{2}-2(1-\mu^{-})\left[\frac{1}{R^{2}}\frac{\partial^{2}w}{\partial \theta^{2}}\frac{\partial^{2}w}{\partial \beta^{2}}-\frac{1}{R^{2}}{\left(\frac{\partial^{2}w}{\partial \theta \partial \beta }\right)}^{2}\right]\right\}dS \end{array}.$$

Integrating with respect to *γ* will yield

(39) $$\begin{array}{c} U_{m}=\frac{1}{2}\frac{E^{+}t_{1}^{3}}{3[1-{\left(\mu^{+}\right)}^{2}]}\iint_{S}\left\{{\left(\frac{1}{R^{2}}\frac{\partial^{2}w}{\partial \theta^{2}}+\frac{\partial^{2}w}{\partial \beta^{2}}\right)}^{2}-2(1-\mu^{+})\left[\frac{1}{R^{2}}\frac{\partial^{2}w}{\partial \theta^{2}}\frac{\partial^{2}w}{\partial \beta^{2}}-\frac{1}{R^{2}}{\left(\frac{\partial^{2}w}{\partial \theta \partial \beta }\right)}^{2}\right]\right\}dS\\ +\frac{1}{2}\frac{E^{-}t_{2}^{3}}{3[1-{\left(\mu^{-}\right)}^{2}]}\iint_{S}\left\{{\left(\frac{1}{R^{2}}\frac{\partial^{2}w}{\partial \theta^{2}}+\frac{\partial^{2}w}{\partial \beta^{2}}\right)}^{2}-2(1-\mu^{-})\left[\frac{1}{R^{2}}\frac{\partial^{2}w}{\partial \theta^{2}}\frac{\partial^{2}w}{\partial \beta^{2}}-\frac{1}{R^{2}}{\left(\frac{\partial^{2}w}{\partial \theta \partial \beta }\right)}^{2}\right]\right\}dS \end{array}.$$

where

(40) $$\iint_{S}\left[\frac{1}{R^{2}}\frac{\partial^{2}w}{\partial \theta^{2}}\frac{\partial^{2}w}{\partial \beta^{2}}-{\left(\frac{1}{R}\frac{\partial^{2}w}{\partial \theta \partial \beta }\right)}^{2}\right]dS=\iint_{S}\frac{1}{R^{2}}\left[\frac{\partial }{\partial \theta }\left(\frac{\partial w}{\partial \theta }\frac{\partial^{2}w}{\partial \beta^{2}}\right)-\frac{\partial }{\partial \beta }\left(\frac{\partial w}{\partial \theta }\frac{\partial^{2}w}{\partial \theta \partial \beta }\right)\right]dS.$$

Due to *dS = Rdθdβ*, and according to the Green formula, Equation (40) is changed as

(41) $$\frac{1}{R}\iint_{S}\left[\frac{\partial }{\partial \theta }\left(\frac{\partial w}{\partial \theta }\frac{\partial^{2}w}{\partial \beta^{2}}\right)-\frac{\partial }{\partial \beta }\left(\frac{\partial w}{\partial \theta }\frac{\partial^{2}w}{\partial \theta \partial \beta }\right)\right]d\theta d\beta =\frac{1}{R}\int_{L}\left(\frac{\partial w}{\partial \theta }\frac{\partial^{2}w}{\partial \beta^{2}}d\beta +\frac{\partial w}{\partial \theta }\frac{\partial^{2}w}{\partial \theta \partial \beta }d\theta \right).$$

Because the four sides of the cylindrical shell are fully fixed,

(42) $${\left(\frac{\partial w}{\partial \theta }\right)}_{L}=0,$$

thus yielding

(43) $$\iint_{S}\left[\frac{1}{R^{2}}\frac{\partial^{2}w}{\partial \theta^{2}}\frac{\partial^{2}w}{\partial \beta^{2}}-{\left(\frac{1}{R}\frac{\partial^{2}w}{\partial \theta \partial \beta }\right)}^{2}\right]Rd\theta d\beta =0.$$

Let

(44) $$D^{*}=\frac{E^{+}t_{1}{}^{3}}{3[1-{\left(\mu^{+}\right)}^{2}]}+\frac{E^{-}t_{2}{}^{3}}{3[1-{\left(\mu^{-}\right)}^{2}]},$$

which is the bending stiffness of a bimodular cylindrical shell in bending; also let the Laplace operator be [31]

(45) $$\nabla^{2}=\frac{1}{R^{2}}\frac{\partial^{2}}{\partial \theta^{2}}+\frac{\partial^{2}}{\partial \beta^{2}},$$

the strain energy *U_m_* may be finally expressed as

(46) $$U_{m}=\frac{D^{*}}{2}\iint_{S}{\left(\nabla^{2}w\right)}^{2}Rd\theta d\beta .$$

Combining Equations (36) and (46), the total strain potential energy which is expressed in terms of the displacement components is finally obtained.

### 6.2. The Ritz Method

For the large deformation problem of the cylindrical shell, the expression of the displacement components is taken as follows:

(47) $$u=\sum_{m}A_{m}u_{m},\;v=\sum_{m}B_{m}v_{m},\;w=\sum_{m}C_{m}w_{m},$$

in which *A_m_*, *B_m_* and *C_m_* are the independent coefficients; and *u_m_*, *v_m_* and *w_m_* are specified functions which are equal to zero on the boundaries. Thus, the displacement components, *u*, *v* and *w*, always satisfy boundary conditions of displacement. As indicated above, the four sides of the cylindrical shell are fully fixed, thus the boundary conditions of displacement give

(48) $${\left(u,\;v,\;w,\;\frac{1}{R}\frac{\partial w}{\partial \theta }\right)}_{\theta =\pm a}=0,{\left(u,\;v,\;w,\;\frac{\partial w}{\partial \beta }\right)}_{\beta =\pm b}=0,$$

where *a* (in radians) is the angle along the *θ* direction and *b* is the length along the *β* direction, please see Figure 1. Further, the displacement *u* and *v* also satisfy the symmetry conditions, that is,

(49) $${\left(u\right)}_{\theta =0}=0,{\left(v\right)}_{\beta =0}=0,$$

this can be easily observed from Figure 1. According to the analysis, the specific expressions of the displacement components can be supposed as follows

(50) $$u=\sum_{m}A_{m}u_{m}=(\theta^{2}-a^{2})(\beta^{2}-b^{2})\theta (A_{0}+A_{1}\theta^{2}+A_{2}\beta^{2}+\cdot \cdot \cdot ),$$

(51) $$v=\sum_{m}B_{m}v_{m}=(\theta^{2}-a^{2})(\beta^{2}-b^{2})\beta (B_{0}+B_{1}\theta^{2}+B_{2}\beta^{2}+\cdot \cdot \cdot )$$

and

(52) $$w=\sum_{m}C_{m}w_{m}={\left(\theta^{2}-a^{2}\right)}^{2}{\left(\beta^{2}-b^{2}\right)}^{2}(C_{0}+C_{1}\theta^{2}+C_{2}\beta^{2}+\cdot \cdot \cdot ).$$

Equations (50)–(52) can satisfy all the boundary conditions of displacement, that is, Equations (48) and (49). Specifically, the term *θ*^2^−*a*^2^ can satisfy *u*, *v* and *w* are zero when *θ = ±a*; similarly, the term *β*^2^−*b*^2^ can satisfy *u*, *v* and *w* are zero when *β = ±b*; the single *θ* and *β* in *u* and *v* are used for satisfying the conditions that *u* and *v* are zero when *θ* and *β* are zero, respectively. The last term in Equations (50)–(52), which is expressed in terms of the series of *θ*^2^ and *β*^2^, is mainly used for the asymptotic in mathematics. In addition, *u*, *v* and *w* are the even functions or odd functions with respect to *θ* and *β*, this feature is also easily observed from Equations (50)–(52).

According to the conclusion from [31,34,35], taking only the first few terms, the variational method of displacement can give relatively satisfactory results. In the next computation, for convenience, the first three undetermined coefficients, *A*_0_, *A*_1_ and *A*_2_, in Equation (50) may be taken; this is the same practice for the three coefficients, *B*_0_, *B*_1_ and *B*_2_, in Equation (51) while the first coefficient *C*_0_ in Equation (52) is taken; and then substituting these displacement formulas into Equations (36) and (46) to obtain the total strain potential energy, which is expressed in terms of *A*_0_, *A*_1_, *A*_2_, *B*_0_, *B*_1_, *B*_2_ and *C*_0_.

According to the variational method of displacement described in Section 3.1 and also considering Equation (9), the following three variational equations will be obtained

(53) $$\frac{\partial U}{\partial A_{m}}=∭Xu_{m}dV+\iint \bar{X}u_{m}dS=0,$$

(54) $$\frac{\partial U}{\partial B_{m}}=∭Yv_{m}dV+\iint \bar{Y}v_{m}dS=0$$

and

(55) $$\frac{\partial U}{\partial C_{m}}=∭Zw_{m}dV+\iint \bar{Z}w_{m}dS=\iint qw_{m}dS,$$

where $\bar{Z}=q$. Via Equations (53) and (54), the equations used for the solution of the coefficients *A*_0_, *A*_1_, *A*_2_, *B*_0_, *B*_1_, and *B*_2_ may be obtained as follows,

(56) $$\left\{ \begin{array}{l} \frac{\partial U}{\partial A_{0}}=0,\frac{\partial U}{\partial A_{1}}=0,\frac{\partial U}{\partial A_{2}}=0\\ \frac{\partial U}{\partial B_{0}}=0,\frac{\partial U}{\partial B_{1}}=0,\frac{\partial U}{\partial B_{2}}=0 \end{array}\right..$$

By using the mathematical software Maple 2021, the coefficients *A*_0_, *A*_1_, *A*_2_, *B*_0_, *B*_1_ and *B*_2_ may be computed, all of which contain *C*_0_ and are listed in Appendix A.

At the same time, when *C*_0_ is considered only, Equation (52) is changed as

(57) $$w=C_{0}w_{0}={\left(\theta^{2}-a^{2}\right)}^{2}{\left(\beta^{2}-b^{2}\right)}^{2}C_{0}.$$

Substituting it into Equation (55) and integrating will yield

(58) $$\frac{\partial U}{\partial C_{0}}=\frac{256}{225}qRa^{5}b^{5}.$$

Because *A*_0_, *A*_1_, *A*_2_, *B*_0_, *B*_1_ and *B*_2_ are now known, substituting them into Equation (58), an equation which contains only *C*_0_ may be obtained. On the other hand, according to Equation (52), and also let the central deflection, that is, the maximum deflection, be *w*_0_ when *θ* and *β* are zero, Equation (52) will give

(59) $$w_{0}=a^{4}b^{4}C_{0}.$$

This means that the central deflection *w*_0_ is indeed the coefficient *C*_0_, only with the difference of the factor *a*^4^*b*^4^. Substituting Equation (59) into Equation (58), the relation of the load vs. central deflection may be finally obtained as follows,

(60) $$\begin{array}{l} \frac{q{\left(Ra\right)}^{4}}{E^{-}t^{4}}[1-{\left(\mu^{-}\right)}^{2}]\\ =\left[\begin{array}{l} {\left(\frac{w_{0}}{t}\right)}^{3}H_{5}(\lambda ,\mu^{-})+{\left(\frac{w_{0}}{t}\right)}^{2}\left(\frac{Ra^{2}}{t}\right)H_{4}(\lambda ,\mu^{-})\\ +\left(\frac{w_{0}}{t}\right)\frac{D^{*}[1-{\left(\mu^{-}\right)}^{2}]}{E^{-}t^{3}}H_{3}(\lambda ,\mu^{-})+\left(\frac{w_{0}}{t}\right){\left(\frac{Ra^{2}}{t}\right)}^{2}H_{2}(\lambda ,\mu^{-}) \end{array}\right]{\left[H_{1}(\lambda ,\mu^{-})\right]}^{-1} \end{array},$$

in which *H*_1_(*λ*, *μ*^−^) to *H*_5_(*λ*, *μ*^−^) are the formulas containing *λ* and *μ*^−^, and they are listed in Appendix B, and *λ = Ra*/*b*.

## 7. Numerical Simulation and Comparison with Theoretical Solution

The numerical simulation is conducted by using the software ABAQUS6.14.4, in which the subroutine UMAT is adopted since, in the existing commercial software, there is no bimodular material model. The whole iteration process will proceed in the following steps:

(i) First, the material is assumed to have a single modulus, and the stress and strain of each element are calculated;
(ii) The principal stress and its direction for each element are thus obtained;
(iii) According to principal stress obtained, the due constitutive relationship between each element is constructed, and the stiffness matrix of each element is collected to form the total stiffness matrix;
(iv) According to the new constitutive relationship, once again, the stress and strain of each element is calculated, and repeat this process;
(v) If reaching the stopping condition, output the final result; otherwise go to (ii).

**Figure 7:**
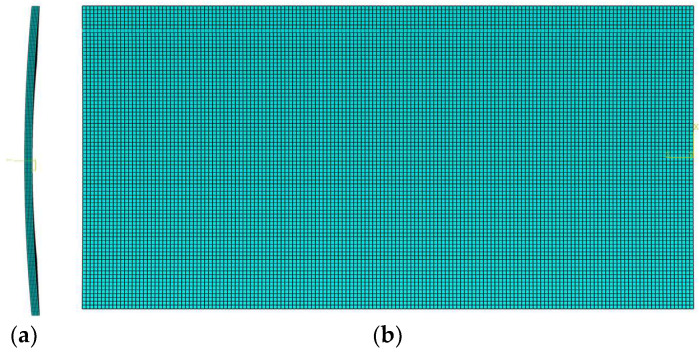
Cylindrical shell model. (**a**) Side elevation; (**b**) plane.

When constructing the computational model of cylindrical shell, the first step is to form the three-dimensional diagram according to the real shape and size. In this study, three groups of model of thin cylindrical shells are used, in which the circumferential length of the shell, 2*Ra*, is 20 m, the longitudinal length, 2*b*, is 40 m and the thickness of the shell, *t*, is 0.2 m (please refer to Figure 1), with different *Ra*^2^/*t* values ranging from 1/2, 1 to 3/2. Figure 7 shows the cylindrical shell model. Note that since the cylindrical shell is relatively shallow, the side elevation and plan of the model, but the three-dimensional diagram, are given in Figure 7.

**Figure 8:**
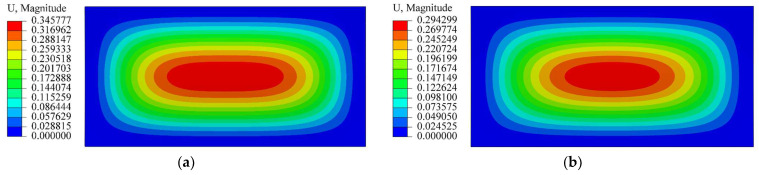
Displacement nephogram when *Ra*^2^/*t =* 1/2 and *q* = 20 Kpa. (**a**) *E*^+^/*E*^−^ = 1; (**b**) *E*^+^/*E*^−^ = 2/3.

**Figure 9:**
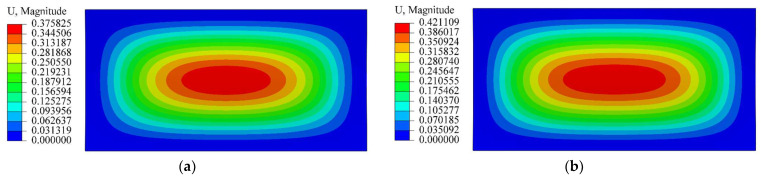
Displacement nephogram when *Ra*^2^/*t =* 1 and *q* = 25 Kpa. (**a**) *E*^+^/*E*^−^ = 1; (**b**) *E*^+^/*E*^−^ = 3/2.

**Figure 10:**
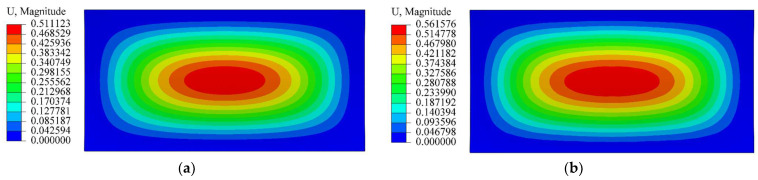
Displacement nephogram when *Ra*^2^/*t =* 3/2 and *q* = 30 Kpa. (**a**) *E*^+^/*E*^−^ = 1; (**b**) *E*^+^/*E*^−^ = 2.

In the numerical simulation, the average modulus, *E*, is taken as 20 Gpa; and the average Poisson’s ratio, *μ*, is taken as 0.3. Five different ratios of *E*^+^/*E*^−^ are selected, and they are 1/2, 2/3, 1, 3/2 and 2 (please refer to the next section). The load intensity ranges from 15 KPa to 30 Kpa, with an interval of 5 Kpa. To compare with the theoretical solution, the boundary conditions in the numerical simulation are also taken as fully fixed. A three-dimensional solid element with 8 nodes, C3D8, is adopted. Some representative displacement nephograms are shown in Figure 8, Figure 9 and Figure 10, in which Figure 8 shows the displacement nephograms when *Ra*^2^/*t =* 1/2 and *q* = 20 Kpa; Figure 9 shows the nephograms when *Ra*^2^/*t =* 1 and *q* = 25 Kpa and Figure 10 shows the nephograms when *Ra*^2^/*t =* 3/2 and *q* = 30 Kpa. From Figure 8, Figure 9 and Figure 10, it is readily apparent that the maximum deflection takes place at the central part of the cylindrical shell; the deflection is gradually reduced as the observation point approaches the shell edge.

Table 1, Table 2 and Table 3 list the central deflection under different bimodular cases (*E*^+^/*E*^−^ = 1/2, 2/3, 1, 3/2 and 2), different loads (*q* = 15 Kpa, 20 Kpa, 25 Kpa and 30 Kpa) and different shapes (*Ra*^2^/*t =* 1/2, 1 and 3/2). The results of the variational method are from Equation (60) and the FEM results are from the numerical simulation. During the mesh division, the shell is divided as 5 or 4 layers along the direction of thickness, with the difference of grid sizes. Specifically, for the case *Ra*^2^/*t =* 1/2, the grid size may be 0.2 m or 0.35 m, thus resulting in 100,000 elements or 32,490 elements, respectively; for the case *Ra*^2^/*t =* 1, the grid size may be 0.25 m or 0.35 m, thus resulting in 64,000 elements or 25,993 elements, respectively; for the case *Ra*^2^/*t =* 3/2, the grid size may be 0.24 m or 0.33 m, thus resulting in 69,305 elements or 29,524 elements, respectively.

By comparing the values of central deflection in Table 1, Table 2 and Table 3, it is easy to see that the values from two different methods are basically consistent but there still exist the differences between them. The variational solution is based on the simplified mechanical model on tension-compression subareas while the numerical simulation is based on the original mechanical model concerning positive–negative signs of principal stresses. Generally, the differences are acceptable, which validates the variational method from the side.

## 8. Results and Discussion

### 8.1. Load vs. Central Deflection

For the convenience of the next analyses, the following quantities are introduced

(61) $$\left\{ \begin{array}{l} \eta =\frac{E^{+}-E^{-}}{E^{+}+E^{-}},E=\frac{E^{+}+E^{-}}{2},\mu =\frac{\mu^{+}+\mu^{-}}{2},\\ P=\frac{q{\left(Ra\right)}^{4}}{E^{-}t^{4}}[1-{\left(\mu^{-}\right)}^{2}],W_{0}=\frac{w_{0}}{t},\\ K=\frac{1+\eta }{1-{\left(\mu^{+}\right)}^{2}}{\left(\frac{t_{1}}{t}\right)}^{3}+\frac{1-\eta }{1-{\left(\mu^{-}\right)}^{2}}{\left(\frac{t_{2}}{t}\right)}^{3}=\frac{3D^{*}}{Et^{3}}=\frac{1-\eta }{1-{\left(\mu^{-}\right)}^{2}}A \end{array}\right.,$$

where *η* is the ratio of the difference of tensile-compressive moduli to the sum of tensile-compressive moduli, and it is obvious that *η* is a small dimensionless quantity which corresponds to the positive (*E*^+^ > *E*^−^) or the negative (*E*^+^ < *E*^−^); *μ* and *E* is the average Poisson’s ratio and average modulus, respectively; *P* and *W*_0_ is the dimensionless load and central deflection, respectively; *K* is the dimensionless bending stiffness, and the relation among *K*, D* and *A* is also shown in Equation (61), and *A* is dimensionless. Thus, Equation (60) now becomes, noting that *H*_1_(*λ*, *μ*^−^) to *H*_5_(*λ*, *μ*^−^) are dimensionless in themselves,

(62) $$P=\frac{W_{0}{}^{3}H_{5}(\lambda ,\mu^{-})+W_{0}{}^{2}\left(\frac{Ra^{2}}{t}\right)H_{4}(\lambda ,\mu^{-})+W_{0}\frac{A}{3}H_{3}(\lambda ,\mu^{-})+W_{0}{\left(\frac{Ra^{2}}{t}\right)}^{2}H_{2}(\lambda ,\mu^{-})}{H_{1}(\lambda ,\mu^{-})},$$

In the bimodular problem, it is generally assumed that the tensile-compressive moduli and tensile-compressive Poisson’s ratios satisfy the familiar relation, *E*^+^/*E*^−^ = *μ*^+^/*μ*^−^ [5]. Therefore, by combining Equation (61), the tensile-compressive moduli and Poisson’s ratios may be computed as

(63) $$\left\{ \begin{array}{l} E^{+}=(1+\eta )E,E^{-}=(1-\eta )E\\ \mu^{+}=(1+\eta )\mu ,\mu^{-}=(1-\eta )\mu \end{array}\right..$$

In addition, the dimensionless tensile-compressive thickness, *t*_1_/*t* and *t*_2_/*t*, are determined via Equation (10) from the previous study [34]. If the *η* value is given in advance, *E*^+^ and *E*^−^ may be expressed in the form of *E* via Equation (63); at the same time, if *μ* is prescribed as 0.3, which is a representative value for most engineering materials (for example, metal), *μ*^+^ and *μ*^−^ may be determined; thus, *t*_1_/*t* and *t*_2_/*t*, as well as *A* and *K* may also be determined via Equation (61). These given values and the corresponding computed values are listed in Table 4.

**Figure 11:**
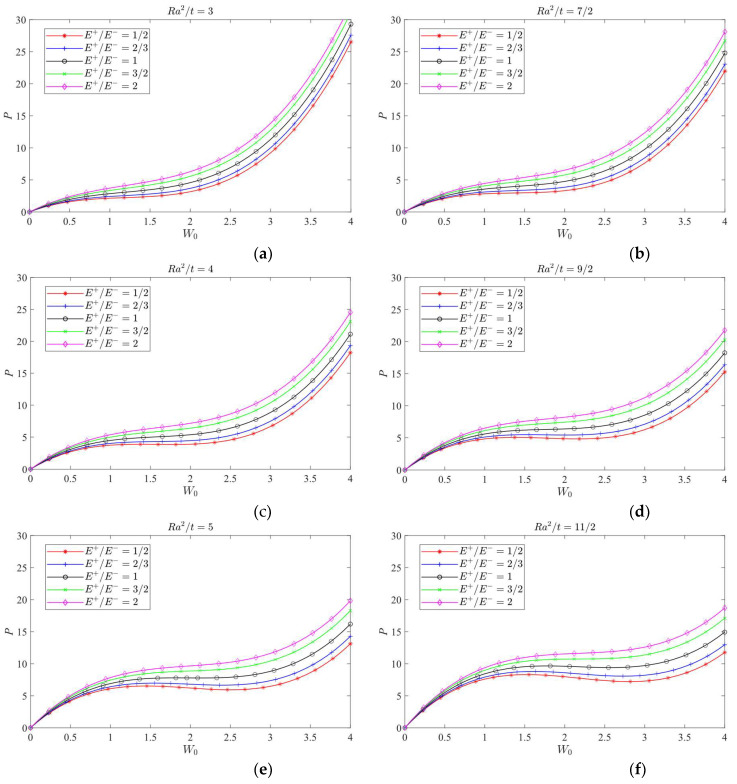
Load vs. central deflection when *λ* = 1/2. (**a**) *Ra*^2^/*t* = 3; (**b**) *Ra*^2^/*t* = 7/2; (**c**) *Ra*^2^/*t* = 4; (**d**) *Ra*^2^/*t* = 9/2; (**e**) *Ra*^2^/*t* = 5; (**f**) *Ra*^2^/*t* = 11/2.

**Figure 12:**
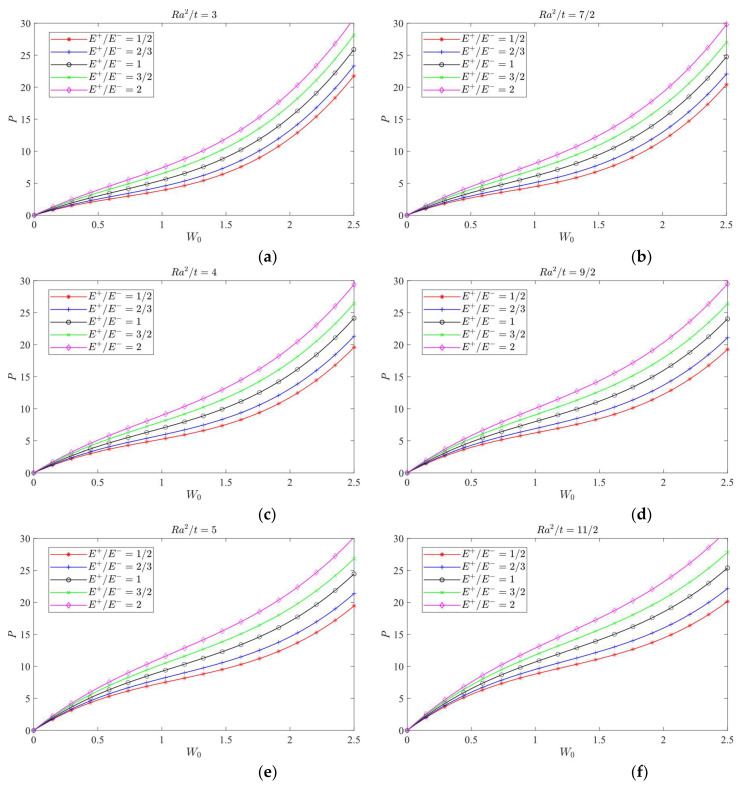
Load vs. central deflection when *λ* = 1. (**a**) *Ra*^2^/*t* = 3; (**b**) *Ra*^2^/*t* = 7/2; (**c**) *Ra*^2^/*t* = 4; (**d**) *Ra*^2^/*t* = 9/2; (**e**) *Ra*^2^/*t* = 5; (**f**) *Ra*^2^/*t* = 11/2.

**Figure 13:**
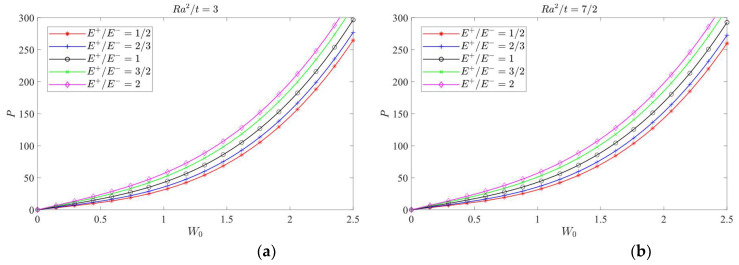

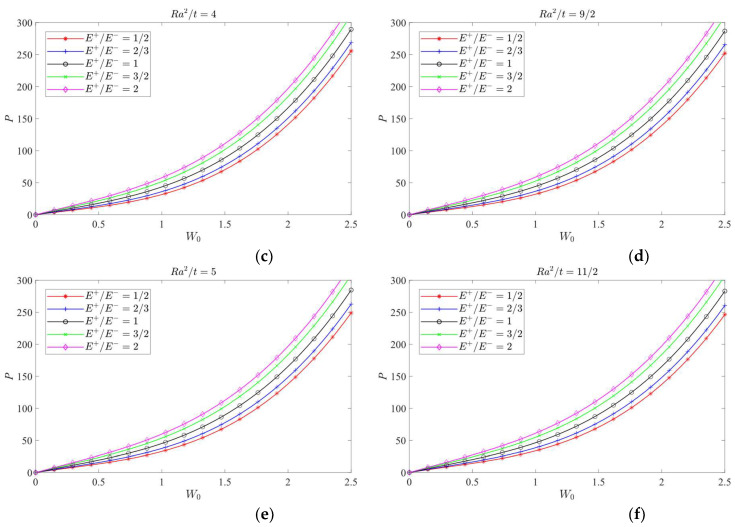
Load vs. central deflection when *λ* = 2. (**a**) *Ra*^2^/*t* = 3; (**b**) *Ra*^2^/*t* = 7/2; (**c**) *Ra*^2^/*t* = 4; (**d**) *Ra*^2^/*t* = 9/2; (**e**) *Ra*^2^/*t* = 5; (**f**) *Ra*^2^/*t* = 11/2.

According to Equation (62) and Table 4, the curves of *P-W*_0_ under different *λ = Ra*/*b* values (1/2, 1 and 2), different *E*^+^/*E*^−^ values (1/2, 2/3, 1, 3/2 and 2) and different *Ra*^2^/*t* values (ranging from 3 to 11/2, with the interval 1/2) may be plotted, as shown in Figure 11, Figure 12 and Figure 13, in which Figure 11 shows the case of *λ* = 1/2, and Figure 12 shows the case of *λ* = 1, and Figure 13 shows the case of *λ* = 2.

Figure 11, Figure 12 and Figure 13 all show that, under the same magnitude of *P*, among the five cases of different moduli, the produced deflection values are in turn, from the least to the most, *E*^+^/*E*^−^ = 2, 3/2, 1, 2/3 and 1/2, indicating that when *E*^+^ > *E*^−^, the central deformation of the cylindrical shell will decrease and when *E*^+^ < *E*^−^, the central deformation of the cylindrical shell will increase. It is obvious that the bimodular effect will change the structural stiffness of the shell, thus resulting in the corresponding change in deformation magnitudes.

For shell structures, the jumping phenomenon is prone to occur in real applications, especially for thin and shallow shells, and the bimodular cylindrical shell presented in this study is no exception. From the perspective of physical phenomena, jumping is a sudden change from one equilibrium state to another, which involves the nonlinear problem and must be solved by nonlinear solving methods. The generation of jumping is influenced by multiple factors, such as initial deflection and uneven stress distribution of shells, but this is not the focus of this study. From Figure 11, the jumping phenomenon is easily observed firstly, which is characterized by the occurrence of inflection point of the curve. However, From Figure 12, the inflection point can be found only in some cases. Further, for Figure 13, no inflection point can be found. This phenomenon indicates that the occurrence of inflection point will depend on several factors—for example, *λ = Ra*/*b* values, different *E*^+^/*E*^−^ values and different *Ra*^2^/*t* values. More in-depth analysis is still needed, especially for the bimodular effect on the jumping phenomenon.

### 8.2. Jumping Phenomenon

**Figure 14:**
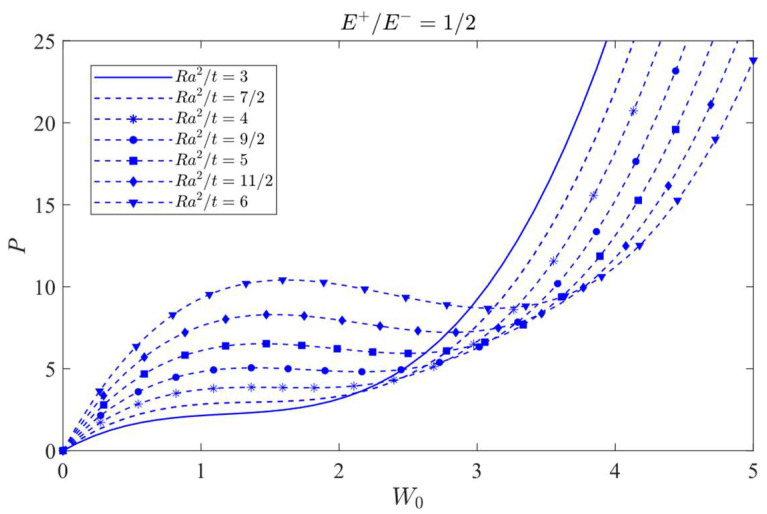
Load vs. central deflection when *λ* = 1/2 and *E*^+^/*E*^−^ = 1/2.

**Figure 15:**
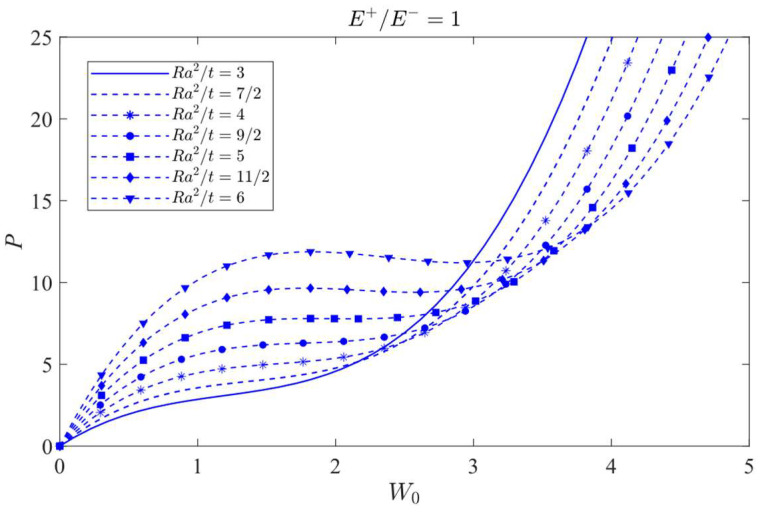
Load vs. central deflection when *λ* = 1/2 and *E*^+^/*E*^−^ = 1.

**Figure 16:**
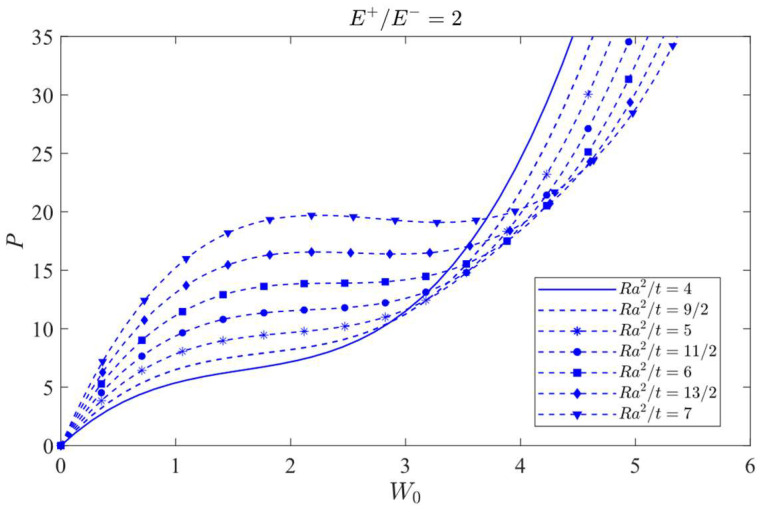
Load vs. central deflection when *λ* = 1/2 and *E*^+^/*E*^−^ = 2.

The attention will be focused on the influence of a bimodular effect on the jumping phenomenon. To this end, the case *λ =* 1/2 is selected as the studied object since in this case, the jumping phenomenon is easily observed. The curves of load vs. central deflection under different *Ra*^2^/*t* values are plotted, as shown in Figure 14, Figure 15 and Figure 16, in which Figure 14 is for the case of *E*^+^/*E*^−^ = 1/2, Figure 15 is for *E*^+^/*E*^−^ = 1, and Figure 16 is for *E*^+^/*E*^−^ = 2.

Figure 14 shows that the jumping phenomenon may occur when *Ra*^2^/*t* is approximately 4, while from Figure 15, the *Ra*^2^/*t* value is approximately 5 and from Figure 16, the *Ra*^2^/*t* value is approximately 6, indicating that under the same *λ* value, the bimodular effect will change the occurrence values of *Ra*^2^/*t* of jumping phenomenon. The smaller *Ra*^2^/*t* value indicates the cylindrical shell is relatively thick while the bigger value shows the shell is relatively thin.

In fact, when the jumping phenomenon takes place, the precise value of *Ra*^2^/*t* may be found via Equation (62). For this reason, differentiating Equation (62) with respect to *W*_0_ and then letting it be zero gives

(64) $$\frac{\partial P}{\partial W_{0}}=\frac{3H_{5}(\lambda ,\mu^{-})W_{0}^{2}+2\left(\frac{Ra^{2}}{t}\right)H_{4}(\lambda ,\mu^{-})W_{0}+\frac{A}{3}H_{3}(\lambda ,\mu^{-})+{\left(\frac{Ra^{2}}{t}\right)}^{2}H_{2}(\lambda ,\mu^{-})}{H_{1}(\lambda ,\mu^{-})}=0$$

When the jumping phenomenon takes place, Equation (64) will give the two real solutions of *W*_0_, this yields

(65) $${\left[2\left(\frac{Ra^{2}}{t}\right)H_{4}(\lambda ,\mu^{-})\right]}^{2}-4\times 3H_{5}(\lambda ,\mu^{-})\times \left[\frac{A}{3}H_{3}(\lambda ,\mu^{-})+{\left(\frac{Ra^{2}}{t}\right)}^{2}H_{2}(\lambda ,\mu^{-})\right]\geq 0,$$

which is further simplified as

(66) $$\frac{Ra^{2}}{t}\geq \frac{\sqrt{\left(H_{4}{}^{2}-3H_{2}H_{5}\right)AH_{3}H_{5}}}{H_{4}{}^{2}-3H_{2}H_{5}}.$$

Via Equation (66), the critical value of *Ra*^2^/*t* under different moduli may be calculated. When *E*^+^/*E*^−^ = 1/2, the critical value of *Ra*^2^/*t* is 3.80; when *E*^+^/*E*^−^ = 1, the critical value of *Ra*^2^/*t* gives 4.89 and when *E*^+^/*E*^−^ = 2, the critical value of *Ra*^2^/*t* changes to be 6.08. These three theoretical values agree with the results observed from Figure 14, Figure 15 and Figure 16.

**Figure 17:**
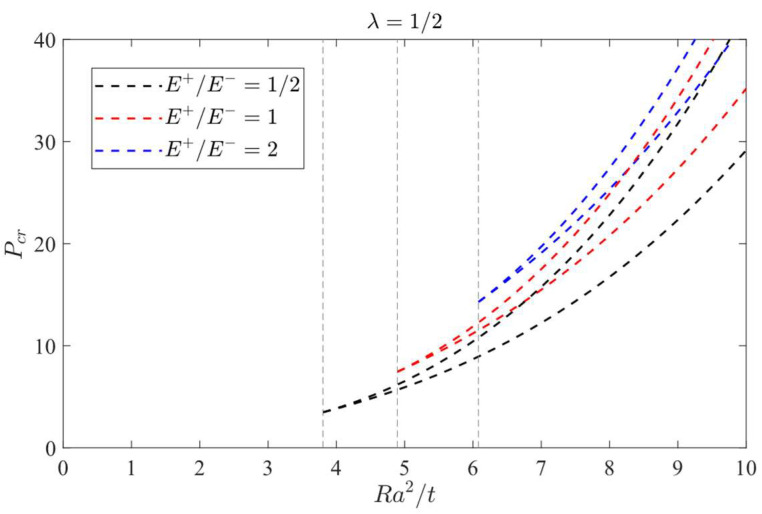
Variation of critical loads with *Ra*^2^/*t* values under different moduli ratios when *λ* = 1/2.

Substituting the two real roots of Equation (64) into Equation (62), the relation of critical load *P_cr_* and the parameter *Ra*^2^/*t* when *λ* = 1/2 may be obtained, which is shown in Figure 17. It is easy to see that from Figure 17, the three groups of curves under different moduli have their own sharp corners, in which the case of *E*^+^/*E*^−^ = 1/2 corresponds to *Ra*^2^/*t* = 3.80, the case of *E*^+^/*E*^−^ = 1 to *Ra*^2^/*t* = 4.89 and the case of *E*^+^/*E*^−^ = 2 to *Ra*^2^/*t* = 6.08.

## 9. Concluding Remarks

In this study, the displacement variational method is applied to solve the large deformation problem of bimodular cylindrical shells. For the convenience of the use of the variational method, the physical equations on the bimodular material model and the geometrical equation under large deformation are derived first; the total strain potential energy is expressed in terms of the displacement component, thus bringing the possibilities for the realization of the Ritz method. Finally, the analytical relationship between load and central deflection is obtained and validated with the numerical simulation. The jumping phenomenon of thin cylindrical shells with a bimodular effect is also discussed. The following three conclusions can be drawn.

(i) Under large deformation and using bimodular materials, the establishment of physical equations and the geometrical equation lays the foundation for the calculation of strain energy and then the application of the variational method.
(ii) The bimodular effect will change the structural stiffness, thus resulting in the corresponding change in the deformation magnitude. Specifically, under the action of the same load, the central deflection of the cylindrical shell will decrease when *E*^+^ > *E*^−^ while the central deflection of the cylindrical shell will increase when *E*^+^ < *E*^−^, in comparison with the central deflection when *E*^+^ = *E*^−^.
(iii) When the shell is relatively thin, the bimodular effect will influence the occurrence of the jumping phenomenon of the cylindrical shell. Specifically, if *λ* = 1/2, that is, the arc length along the θ direction is half of line length along the *β* direction, the shell is a long shell, when *E*^+^/*E*^−^ = 1/2, the critical value of *Ra*^2^/*t* is 3.80; when *E*^+^/*E*^−^ = 1, the critical value of *Ra*^2^/*t* gives 4.89 and when *E*^+^/*E*^−^ = 2, the critical value of *Ra*^2^/*t* changes to be 6.08.

In addition, the occurrence of the jumping phenomenon depends on many factors, including *λ = Ra*/*b* values (is the cylindrical shell relatively long or short?), different *Ra*^2^/*t* values (is the cylindrical shell relatively thick or thin?), different load types (uniformly distributed loads or concentrated force, or both) and all kinds of boundary conditions (fully fixed, simply supported or the mix). Limited to the use of the variational method, the bimodular cylindrical shell in this study is fully fixed at its four sides, and the load considered is also uniformly distributed load. In practical problems, the load and boundary conditions may be diverse. More investigations are still needed, especially for the relatively thin cylindrical shells when the above factors are superimposed together.

If shell structures are composed of the materials with a relatively obvious bimodular effect; or even if the material has no obvious bimodular effect, the analytical results obtained in this study may be used for the refined analysis and optimized design. Specifically, via the analytical relationship between load and central deflection, it is readily known for the designers what will be the maximum deflection under a certain design load, which is often required in the design codes and specifications.

The limitations of this study exist in the following two aspects: the cylindrical shell is a particular case of a shell with curvature in both directions, so this general case should be studied in future studies; this study does not incorporate the effects of a snap-through phenomenon due to instability, so this effect should also be incorporated in future studies.

## Figures and Tables

**Table 1:** Central deflections under different bimodular cases and loads (*Ra*^2^/*t =* 1/2).

*E*^+^/*E*^−^	q (Kpa)	Central Deflection *w*_0_ (m)	
		Variational Method	FEM
1/2	15	0.262811	0.227548
	20	0.297710	0.290979
	25	0.326251	0.340906
	30	0.350656	0.382729
2/3	15	0.267203	0.230800
	20	0.304338	0.294299
	25	0.334660	0.344613
	30	0.360554	0.385766
1	15	0.277442	0.295023
	20	0.318139	0.345777
	25	0.351337	0.384648
	30	0.379657	0.417361
3/2	15	0.294951	0.271896
	20	0.339607	0.318476
	25	0.376050	0.355947
	30	0.407135	0.388520
2	15	0.312865	0.275682
	20	0.360583	0.325684
	25	0.399556	0.361290
	30	0.432812	0.393627

**Table 2:** Central deflections under different bimodular cases and loads (*Ra*^2^/*t =* 1).

*E*^+^/*E*^−^	q (Kpa)	Central Deflection *w*_0_ (m)	
		Variational Method	FEM
1/2	15	0.294340	0.217232
	20	0.331284	0.326770
	25	0.361030	0.404759
	30	0.386234	0.462075
2/3	15	0.297410	0.221017
	20	0.336883	0.320205
	25	0.368587	0.395324
	30	0.395397	0.450756
1	15	0.306059	0.223635
	20	0.349428	0.308476
	25	0.384217	0.375825
	30	0.413595	0.428147
3/2	15	0.322687	0.296831
	20	0.370170	0.368591
	25	0.408309	0.421109
	30	0.440528	0.461153
2	15	0.340483	0.327177
	20	0.391018	0.375792
	25	0.431690	0.428531
	30	0.466087	0.471214

**Table 3:** Central deflections under different bimodular cases and loads (*Ra*^2^/*t =* 3/2).

*E*^+^/*E*^−^	q (Kpa)	Central Deflection *w*_0_ (m)	
		Variational Method	FEM
1/2	15	0.324920	0.214879
	20	0.364591	0.269926
	25	0.395850	0.450152
	30	0.422018	0.545194
2/3	15	0.326456	0.243589
	20	0.369030	0.288025
	25	0.402457	0.470796
	30	0.430367	0.572830
1	15	0.333323	0.277248
	20	0.380188	0.306887
	25	0.416937	0.392184
	30	0.447571	0.511123
3/2	15	0.349096	0.290966
	20	0.400188	0.418038
	25	0.440379	0.500748
	30	0.473923	0.558026
2	15	0.366912	0.346429
	20	0.420965	0.464555
	25	0.463658	0.519060
	30	0.499369	0.561576

**Table 4:** The computed values in the relation of load vs. central deflection.

*η*	*E* ^+^	*E* ^−^	*E*^+^/*E*^−^	*μ* ^+^	*μ* ^−^	*t*_1_/*t*	*t*_2_/*t*	*A*	*K*
−1/3	2/3*E*	4/3*E*	1/2	0.2	0.4	0.6019	0.3981	0.1585	0.2516
−1/5	4/5*E*	6/5*E*	2/3	0.24	0.36	0.5603	0.4397	0.1933	0.2665
0	*E*	*E*	1	0.3	0.3	0.5	0.5	0.25	0.2747
1/5	6/5*E*	4/5*E*	3/2	0.36	0.24	0.4397	0.5603	0.3140	0.2665
1/3	4/3*E*	2/3*E*	2	0.4	0.2	0.3981	0.6019	0.3623	0.2516

## Data Availability

Not applicable.

## Appendix A

*A*_0_, *A*_1_, *A*_2_, *B*_0_, *B*_1_ and *B*_2_ are listed as follows, in which, for brevity, let *μ*^−^ be *μ*.

$$\begin{array}{ll} A_{0} & =4a^{2}b^{4}C_{0}[407546092800R^{12}a^{14}b^{2}\mu^{3}C_{0}-911292560640R^{12}a^{14}b^{2}\mu^{2}C_{0}-539142885120R^{10}a^{12}b^{4}\mu^{4}C_{0}+599946842880R^{12}a^{14}b^{2}\mu C_{0}\\ & +1942013337600R^{10}a^{12}b^{4}\mu^{3}C_{0}+49494829440R^{8}a^{10}b^{6}\mu^{5}C_{0}-96200375040R^{12}a^{14}b^{2}C_{0}-15579319956480R^{10}a^{12}b^{4}\mu^{2}C_{0}\\ & -344730419712R^{8}a^{10}b^{6}\mu^{4}C_{0}-580070400R^{6}a^{8}b^{8}\mu^{6}C_{0}+18115319470080R^{10}a^{12}b^{4}\mu C_{0}+19504799872768R^{8}a^{10}b^{6}\mu^{3}C_{0}\\ & +6614285568R^{6}a^{8}b^{8}\mu^{5}C_{0}-3938869966080R^{10}a^{12}b^{4}C_{0}-45771071786496R^{8}a^{10}b^{6}\mu^{2}C_{0}-3278140982656R^{6}a^{8}b^{8}\mu^{4}C_{0}\\ & +45813062924160R^{8}a^{10}b^{6}\mu C_{0}+10383747818752R^{6}a^{8}b^{8}\mu^{3}C_{0}+49243232640R^{4}a^{6}b^{10}\mu^{5}C_{0}+28156998870R^{11}a^{10}\mu^{4}\\ & -16225135790080R^{8}a^{10}b^{6}C_{0}-36675115531264R^{6}a^{8}b^{8}\mu^{2}C_{0}-292409951232R^{4}a^{6}b^{10}\mu^{4}C_{0}-28156998870R^{11}a^{10}\mu^{3}\\ & -19284962697R^{9}a^{8}b^{2}\mu^{5}+47114565987840R^{6}a^{8}b^{8}\mu C_{0}+5822261073664R^{4}a^{6}b^{10}\mu^{3}C_{0}-238762193670R^{11}a^{10}\mu^{2}\\ & +32857859034R^{9}a^{8}b^{2}\mu^{4}+274173900R^{7}a^{6}b^{4}\mu^{6}-20532583547520R^{6}a^{8}b^{8}C_{0}-10724250084352R^{4}a^{6}b^{10}\mu^{2}C_{0}\\ & -118378592640R^{2}a^{4}b^{12}\mu^{4}C_{0}+449367388470R^{11}a^{10}\mu -1039468834404R^{9}a^{8}b^{2}\mu^{3}-1490382894R^{7}a^{6}b^{4}\mu^{5}\\ & +14252499632000R^{4}a^{6}b^{10}\mu C_{0}+341881182720R^{2}a^{4}b^{12}\mu^{3}C_{0}-210605194800R^{11}a^{10}-901286179794R^{9}a^{8}b^{2}\mu^{2}\\ & +1219386471303R^{7}a^{6}b^{4}\mu^{4}-9107343902720R^{4}a^{6}b^{10}C_{0}-1411545864960R^{2}a^{4}b^{12}\mu^{2}C_{0}+12071149991901R^{9}a^{8}b^{2}\mu \\ & -1062305604360R^{7}a^{6}b^{4}\mu^{3}-20059586547R^{5}a^{4}b^{6}\mu^{5}+2270962552320R^{2}a^{4}b^{12}\mu C_{0}+21794572800a^{2}b^{14}\mu^{3}C_{0}\\ & -10143967874040R^{9}a^{8}b^{2}-12510118162542R^{7}a^{6}b^{4}\mu^{2}+78825175572R^{5}a^{4}b^{6}\mu^{4}-1082919277440R^{2}a^{4}b^{12}C_{0}\\ & -65383718400a^{2}b^{14}\mu^{2}C_{0}+31196316245094R^{7}a^{6}b^{4}\mu -514572661746R^{5}a^{4}b^{6}\mu^{3}+65383718400a^{2}b^{14}\mu C_{0}\\ & -21729490483701R^{7}a^{6}b^{4}-5899167065220R^{5}a^{4}b^{6}\mu^{2}+25746613893R^{3}a^{2}b^{8}\mu^{4}-21794572800a^{2}b^{14}C_{0}\\ & +19674318642693R^{5}a^{4}b^{6}\mu +102264222060R^{3}a^{2}b^{8}\mu^{3}-13319344504752R^{5}a^{4}b^{6}-1906630075350R^{3}a^{2}b^{8}\mu^{2}\\ & +3403481028948R^{3}a^{2}b^{8}\mu +32768075340Rb^{10}\mu^{3}-1624861789551R^{3}a^{2}b^{8}-98304226020Rb^{10}\mu^{2}\\ & +98304226020Rb^{10}\mu -32768075340Rb^{10}]/99099R[2910600R^{12}a^{12}\mu^{3}-8731800R^{12}a^{12}\mu^{2}-1897455R^{10}a^{10}b^{2}\mu^{4}\\ & +8731800R^{12}a^{12}\mu +15901200R^{10}a^{10}b^{2}\mu^{3}+29050R^{8}a^{8}b^{4}\mu^{5}-2910600R^{12}a^{12}-170432850R^{10}a^{10}b^{2}\mu^{2}-1480150R^{8}a^{8}b^{4}\mu^{4}\\ & +300751920R^{10}a^{10}b^{2}\mu +143573920R^{8}a^{8}b^{4}\mu^{3}+16600R^{6}a^{6}b^{6}\mu^{5}-144322815R^{10}a^{10}b^{2}-648770080R^{8}a^{8}b^{4}\mu^{2}\\ & -4259394R^{6}a^{6}b^{6}\mu^{4}+1692606790R^{8}a^{8}b^{4}\mu +102263512R^{6}a^{6}b^{6}\mu^{3}+29050R^{4}a^{4}b^{8}\mu^{5}-1185959530R^{8}a^{8}b^{4}\\ & -1532919728R^{6}a^{6}b^{6}\mu^{2}-1480150R^{4}a^{4}b^{8}\mu^{4}+3190654040R^{6}a^{6}b^{6}\mu +143573920R^{4}a^{4}b^{8}\mu^{3}-2094893830R^{6}a^{6}b^{6}\\ & -648770080R^{4}a^{4}b^{8}\mu^{2}-1897455R^{2}a^{2}b^{10}\mu^{4}+1692606790R^{4}a^{4}b^{8}\mu +15901200R^{2}a^{2}b^{10}\mu^{3}-1185959530R^{4}a^{4}b^{8}\\ & -170432850R^{2}a^{2}b^{10}\mu^{2}+300751920R^{2}a^{2}b^{10}\mu +2910600b^{12}\mu^{3}-144322815R^{2}a^{2}b^{10}-8731800b^{12}\mu^{2}+8731800b^{12}\mu -2910600b^{12}] \end{array}$$

$$\begin{array}{ll} A_{1} & =-8b^{6}C_{0}[1170892800R^{12}a^{14}\mu^{3}C_{0}-2575964160R^{12}a^{14}\mu^{2}C_{0}-1717330560R^{10}a^{12}b^{2}\mu^{4}C_{0}+1639249920R^{12}a^{14}\mu C_{0}+5338924800R^{10}a^{12}b^{2}\mu^{3}C_{0}\\ & +39459200R^{8}a^{10}b^{4}\mu^{5}C_{0}-234178560R^{12}a^{14}C_{0}-30261623040R^{10}a^{12}b^{2}\mu^{2}C_{0}-554781184R^{8}a^{10}b^{4}\mu^{4}C_{0}+35100975360R^{10}a^{12}b^{2}\mu C_{0}\\ & +33228491392R^{8}a^{10}b^{4}\mu^{3}C_{0}+6841856R^{6}a^{8}b^{6}\mu^{5}C_{0}-8460946560R^{10}a^{12}b^{2}C_{0}-88011920768R^{8}a^{10}b^{4}\mu^{2}C_{0}-3448479488R^{6}a^{8}b^{6}\mu^{4}C_{0}\\ & +109728729600R^{8}a^{10}b^{4}\mu C_{0}+16246501888R^{6}a^{8}b^{6}\mu^{3}C_{0}+52438400R^{4}a^{6}b^{8}\mu^{5}C_{0}-48058665600R^{8}a^{10}b^{4}C_{0}-93467685376R^{6}a^{8}b^{6}\mu^{2}C_{0}\\ & -372031744R^{4}a^{6}b^{8}\mu^{4}C_{0}-4204629R^{9}a^{8}\mu^{5}+148592168960R^{6}a^{8}b^{6}\mu C_{0}+9103828608R^{4}a^{6}b^{8}\mu^{3}C_{0}-301798926R^{9}a^{8}\mu^{4}\\ & -79542081280R^{6}a^{8}b^{6}C_{0}-31255516544R^{4}a^{6}b^{8}\mu^{2}C_{0}-135703680R^{2}a^{4}b^{10}\mu^{4}C_{0}-494277498R^{9}a^{8}\mu^{3}+5021016R^{7}a^{6}b^{2}\mu^{5}\\ & +67284474880R^{4}a^{6}b^{8}\mu C_{0}+795782400R^{2}a^{4}b^{10}\mu^{3}C_{0}+825041646R^{9}a^{8}\mu^{2}+529080123R^{7}a^{6}b^{2}\mu^{4}-44813193600R^{4}a^{6}b^{8}C_{0}\\ & -6641402880R^{2}a^{4}b^{10}\mu^{2}C_{0}+9131987007R^{9}a^{8}\mu +216035820R^{7}a^{6}b^{2}\mu^{3}-10339329R^{5}a^{4}b^{4}\mu^{5}+11438273280R^{2}a^{4}b^{10}\mu C_{0}\\ & +110073600a^{2}b^{12}\mu^{3}C_{0}-9156747600R^{9}a^{8}-10603564686R^{7}a^{6}b^{2}\mu^{2}+13600158R^{5}a^{4}b^{4}\mu^{4}-5456949120R^{2}a^{4}b^{10}C_{0}\\ & -330220800a^{2}b^{12}\mu^{2}C_{0}+25186322304R^{7}a^{6}b^{2}\mu +928501002R^{5}a^{4}b^{4}\mu^{3}+330220800a^{2}b^{12}\mu C_{0}-18748506777R^{7}a^{6}b^{2}\\ & -5191834362R^{5}a^{4}b^{4}\mu^{2}-10008999R^{3}a^{2}b^{6}\mu^{4}-110073600a^{2}b^{12}C_{0}+15130553295R^{5}a^{4}b^{4}\mu +123873750R^{3}a^{2}b^{6}\mu^{3}-10870480764R^{5}a^{4}b^{4}\\ & -1543962420R^{3}a^{2}b^{6}\mu^{2}+2756339586R^{3}a^{2}b^{6}\mu +26756730Rb^{8}\mu^{3}-1326241917R^{3}a^{2}b^{6}-80270190Rb^{8}\mu^{2}+80270190Rb^{8}\mu \\ & -26756730Rb^{8}]/429R[2910600R^{12}a^{12}\mu^{3}-8731800R^{12}a^{12}\mu^{2}-1897455R^{10}a^{10}b^{2}\mu^{4}+8731800R^{12}a^{12}\mu +15901200R^{10}a^{10}b^{2}\mu^{3}\\ & +29050R^{8}a^{8}b^{4}\mu^{5}-2910600R^{12}a^{12}-170432850R^{10}a^{10}b^{2}\mu^{2}-1480150R^{8}a^{8}b^{4}\mu^{4}+300751920R^{10}a^{10}b^{2}\mu +143573920R^{8}a^{8}b^{4}\mu^{3}\\ & +16600R^{6}a^{6}b^{6}\mu^{5}-144322815R^{10}a^{10}b^{2}-648770080R^{8}a^{8}b^{4}\mu^{2}-4259394R^{6}a^{6}b^{6}\mu^{4}+1692606790R^{8}a^{8}b^{4}\mu +102263512R^{6}a^{6}b^{6}\mu^{3}\\ & +29050R^{4}a^{4}b^{8}\mu^{5}-1185959530R^{8}a^{8}b^{4}-1532919728R^{6}a^{6}b^{6}\mu^{2}-1480150R^{4}a^{4}b^{8}\mu^{4}+3190654040R^{6}a^{6}b^{6}\mu +143573920R^{4}a^{4}b^{8}\mu^{3}\\ & -2094893830R^{6}a^{6}b^{6}-648770080R^{4}a^{4}b^{8}\mu^{2}-1897455R^{2}a^{2}b^{10}\mu^{4}+1692606790R^{4}a^{4}b^{8}\mu +15901200R^{2}a^{2}b^{10}\mu^{3}-1185959530R^{4}a^{4}b^{8}\\ & -170432850R^{2}a^{2}b^{10}\mu^{2}+300751920R^{2}a^{2}b^{10}\mu +2910600b^{12}\mu^{3}-144322815R^{2}a^{2}b^{10}-8731800b^{12}\mu^{2}+8731800b^{12}\mu -2910600b^{12}] \end{array}$$

$$\begin{array}{ll} A_{2} & =-4a^{2}b^{2}C_{0}[10090080000R^{11}a^{14}b^{2}\mu^{3}C_{0}-32691859200R^{11}a^{14}b^{2}\mu^{2}C_{0}-25592985600R^{9}a^{12}b^{4}\mu^{4}C_{0}+35113478400R^{11}a^{14}b^{2}\mu C_{0}+55871854080R^{9}a^{12}b^{4}\mu^{3}C_{0}\\ & +6732289920R^{7}a^{10}b^{6}\mu^{5}C_{0}-12511699200R^{11}a^{14}b^{2}C_{0}-534334993920R^{9}a^{12}b^{4}\mu^{2}C_{0}-29057364480R^{7}a^{10}b^{6}\mu^{4}C_{0}-82867200R^{5}a^{8}b^{8}\mu^{6}C_{0}\\ & +1104016704000R^{9}a^{12}b^{4}\mu C_{0}+1167536967680R^{7}a^{10}b^{6}\mu^{3}C_{0}+757927680R^{5}a^{8}b^{8}\mu^{5}C_{0}-599960578560R^{9}a^{12}b^{4}C_{0}-1533653195520R^{7}a^{10}b^{6}\mu^{2}C_{0}\\ & -438345448576R^{5}a^{8}b^{8}\mu^{4}C_{0}+3710291080320R^{7}a^{10}b^{6}\mu C_{0}+619593390848R^{5}a^{8}b^{8}\mu^{3}C_{0}+6662401920R^{3}a^{6}b^{10}\mu^{5}C_{0}+4414860450R^{10}a^{10}\mu^{4}\\ & -4122350155520R^{7}a^{10}b^{6}C_{0}-3363679726592R^{5}a^{8}b^{8}\mu^{2}C_{0}-32218974720R^{3}a^{6}b^{10}\mu^{4}C_{0}-4414860450R^{10}a^{10}\mu^{3}-2911033125R^{8}a^{8}b^{2}\mu^{5}\\ & +6499828303360R^{5}a^{8}b^{8}\mu C_{0}+1796473574400R^{3}a^{6}b^{10}\mu^{3}C_{0}-13571608050R^{10}a^{10}\mu^{2}+12755032290R^{8}a^{8}b^{2}\mu^{4}+39167700R^{6}a^{6}b^{4}\mu^{6}\\ & -3839509173120R^{5}a^{8}b^{8}C_{0}-1543860046080R^{3}a^{6}b^{10}\mu^{2}C_{0}-51233212800Ra^{4}b^{12}\mu^{4}C_{0}+22728355650R^{10}a^{10}\mu -191165934960R^{8}a^{8}b^{2}\mu^{3}\\ & -305102226R^{6}a^{6}b^{4}\mu^{5}+22667164800R^{3}a^{6}b^{10}\mu C_{0}+84761994240Ra^{4}b^{12}\mu^{3}C_{0}-9156747600R^{10}a^{10}-47526889410R^{8}a^{8}b^{2}\mu^{2}\\ & +194205032307R^{6}a^{6}b^{4}\mu^{4}-249724120320R^{3}a^{6}b^{10}C_{0}-18965802240Ra^{4}b^{12}\mu^{2}C_{0}+634179591045R^{8}a^{8}b^{2}\mu \\ & -264847155144R^{6}a^{6}b^{4}\mu^{3}-3005649075R^{4}a^{4}b^{6}\mu^{5}-11421527040Ra^{4}b^{12}\mu C_{0}-405330765840R^{8}a^{8}b^{2}+130504048818R^{6}a^{6}b^{4}\mu^{2}\\ & +12988273584R^{4}a^{4}b^{6}\mu^{4}-3141452160Ra^{4}b^{12}C_{0}+1960602694050R^{6}a^{6}b^{4}\mu -1075250076186R^{4}a^{4}b^{6}\mu^{3}-1823546629905R^{6}a^{6}b^{4}\\ & -120503628960R^{4}a^{4}b^{6}\mu^{2}+24722723025R^{2}a^{2}b^{8}\mu^{4}+2783017428477R^{4}a^{4}b^{6}\mu -21559978440R^{2}a^{2}b^{8}\mu^{3}-1597246347840R^{4}a^{4}b^{6}\\ & -225825810210R^{2}a^{2}b^{8}\mu^{2}+417440663640R^{2}a^{2}b^{8}\mu +3924320400b^{10}\mu^{3}-194777598015R^{2}a^{2}b^{8}-11772961200b^{10}\mu^{2}+11772961200b^{10}\mu \\ & -3924320400b^{10}]/14157[2910600R^{12}a^{12}\mu^{3}8731800R^{12}a^{12}\mu^{2}-1897455R^{10}a^{10}b^{2}\mu^{4}+8731800R^{12}a^{12}\mu +15901200R^{10}a^{10}b^{2}\mu^{3}+29050R^{8}a^{8}b^{4}\mu^{5}\\ & -2910600R^{12}a^{12}-170432850R^{10}a^{10}b^{2}\mu^{2}-1480150R^{8}a^{8}b^{4}\mu^{4}+300751920R^{10}a^{10}b^{2}\mu +143573920R^{8}a^{8}b^{4}\mu^{3}+16600R^{6}a^{6}b^{6}\mu^{5}\\ & -144322815R^{10}a^{10}b^{2}-648770080R^{8}a^{8}b^{4}\mu^{2}-4259394R^{6}a^{6}b^{6}\mu^{4}+1692606790R^{8}a^{8}b^{4}\mu +102263512R^{6}a^{6}b^{6}\mu^{3}+29050R^{4}a^{4}b^{8}\mu^{5}\\ & -1185959530R^{8}a^{8}b^{4}-1532919728R^{6}a^{6}b^{6}\mu^{2}-1480150R^{4}a^{4}b^{8}\mu^{4}+3190654040R^{6}a^{6}b^{6}\mu +143573920R^{4}a^{4}b^{8}\mu^{3}-2094893830R^{6}a^{6}b^{6}\\ & -648770080R^{4}a^{4}b^{8}\mu^{2}-1897455R^{2}a^{2}b^{10}\mu^{4}+1692606790R^{4}a^{4}b^{8}\mu +15901200R^{2}a^{2}b^{10}\mu^{3}-1185959530R^{4}a^{4}b^{8}-170432850R^{2}a^{2}b^{10}\mu^{2}\\ & +300751920R^{2}a^{2}b^{10}\mu +2910600b^{12}\mu^{3}-144322815R^{2}a^{2}b^{10}-8731800b^{12}\mu^{2}+8731800b^{12}\mu -2910600b^{12}] \end{array}$$

$$\begin{array}{ll} B_{0} & =4a^{4}b^{2}C_{0}[21794572800R^{12}a^{14}b^{2}\mu^{3}C_{0}-65383718400R^{12}a^{14}b^{2}\mu^{2}C_{0}-118378592640R^{10}a^{12}b^{4}\mu^{4}C_{0}+65383718400R^{12}a^{14}b^{2}\mu C_{0}+341881182720R^{10}a^{12}b^{4}\mu^{3}C_{0}\\ & +49243232640R^{8}a^{10}b^{6}\mu^{5}C_{0}-21794572800R^{12}a^{14}b^{2}C_{0}-1411545864960R^{10}a^{12}b^{4}\mu^{2}C_{0}-292409951232R^{8}a^{10}b^{6}\mu^{4}C_{0}-580070400R^{6}a^{8}b^{8}\mu^{6}C_{0}\\ & +2270962552320R^{10}a^{12}b^{4}\mu C_{0}+5822261073664R^{8}a^{10}b^{6}\mu^{3}C_{0}+6614285568R^{6}a^{8}b^{8}\mu^{5}C_{0}-1082919277440R^{10}a^{12}b^{4}C_{0}-10724250084352R^{8}a^{10}b^{6}\mu^{2}C_{0}\\ & -3278140982656R^{6}a^{8}b^{8}\mu^{4}C_{0}+14252499632000R^{8}a^{10}b^{6}\mu C_{0}+10383747818752R^{6}a^{8}b^{8}\mu^{3}C_{0}+49494829440R^{4}a^{6}b^{10}\mu^{5}C_{0}+32768075340R^{11}a^{10}\mu^{4}\\ & -9107343902720R^{8}a^{10}b^{6}C_{0}-36675115531264R^{6}a^{8}b^{8}\mu^{2}C_{0}-344730419712R^{4}a^{6}b^{10}\mu^{4}C_{0}-98304226020R^{11}a^{10}\mu^{3}-21410636247R^{9}a^{8}b^{2}\mu^{5}\\ & +47114565987840R^{6}a^{8}b^{8}\mu C_{0}+19504799872768R^{4}a^{6}b^{10}\mu^{3}C_{0}+98304226020R^{11}a^{10}\mu^{2}+149421472200R^{9}a^{8}b^{2}\mu^{4}+274173900R^{7}a^{6}b^{4}\mu^{6}\\ & -20532583547520R^{6}a^{8}b^{8}C_{0}-45771071786496R^{4}a^{6}b^{10}\mu^{2}C_{0}-539142885120R^{2}a^{4}b^{12}\mu^{4}C_{0}-32768075340R^{11}a^{10}\mu -1812315575070R^{9}a^{8}b^{2}\mu^{3}\\ & -2773007094R^{7}a^{6}b^{4}\mu^{5}+45813062924160R^{4}a^{6}b^{10}\mu C_{0}+1942013337600R^{2}a^{4}b^{12}\mu^{3}C_{0}+3309166528668R^{9}a^{8}b^{2}\mu^{2}+1493221603731R^{7}a^{6}b^{4}\mu^{4}\\ & -16225135790080R^{4}a^{6}b^{10}C_{0}-15579319956480R^{2}a^{4}b^{12}\mu^{2}C_{0}-1672019039691R^{9}a^{8}b^{2}\mu -5735970699888R^{7}a^{6}b^{4}\mu^{3}-22423097697R^{5}a^{4}b^{6}\mu^{5}\\ & +18115319470080R^{2}a^{4}b^{12}\mu C_{0}+407546092800a^{2}b^{14}\mu^{3}C_{0}+47157250140R^{9}a^{8}b^{2}+15597728830398R^{7}a^{6}b^{4}\mu^{2}+150818173506R^{5}a^{4}b^{6}\mu^{4}\\ & -3938869966080R^{2}a^{4}b^{12}C_{0}-911292560640a^{2}b^{14}\mu^{2}C_{0}-13400942687418R^{7}a^{6}b^{4}\mu -12088508602170R^{5}a^{4}b^{6}\mu^{3}+599946842880a^{2}b^{14}\mu C_{0}\\ & +2048461786371R^{7}a^{6}b^{4}+26702765441352R^{5}a^{4}b^{6}\mu^{2}+262865746143R^{3}a^{2}b^{8}\mu^{4}-96200375040a^{2}b^{14}C_{0}-20909016301173R^{5}a^{4}b^{6}\mu \\ & -835570461726R^{3}a^{2}b^{8}\mu^{3}+3278936642982R^{5}a^{4}b^{6}+9408625942716R^{3}a^{2}b^{8}\mu^{2}-10176825733074R^{3}a^{2}b^{8}\mu -182448195930Rb^{10}\mu^{3}\\ & +1340904505941R^{3}a^{2}b^{8}+393053390730Rb^{10}\mu^{2}-238762193670Rb^{10}\mu +28156998870Rb^{10}]/99099[2910600R^{12}a^{12}\mu^{3}-8731800R^{12}a^{12}\mu^{2}\\ & -1897455R^{10}a^{10}b^{2}\mu^{4}+8731800R^{12}a^{12}\mu +15901200R^{10}a^{10}b^{2}\mu^{3}+29050R^{8}a^{8}b^{4}\mu^{5}-2910600R^{12}a^{12}-170432850R^{10}a^{10}b^{2}\mu^{2}-1480150R^{8}a^{8}b^{4}\mu^{4}\\ & +300751920R^{10}a^{10}b^{2}\mu +143573920R^{8}a^{8}b^{4}\mu^{3}+16600R^{6}a^{6}b^{6}\mu^{5}-144322815R^{10}a^{10}b^{2}-648770080R^{8}a^{8}b^{4}\mu^{2}-4259394R^{6}a^{6}b^{6}\mu^{4}+1692606790R^{8}a^{8}b^{4}\mu \\ & +102263512R^{6}a^{6}b^{6}\mu^{3}+29050R^{4}a^{4}b^{8}\mu^{5}-1185959530R^{8}a^{8}b^{4}-1532919728R^{6}a^{6}b^{6}\mu^{2}-1480150R^{4}a^{4}b^{8}\mu^{4}+3190654040R^{6}a^{6}b^{6}\mu \\ & +143573920R^{4}a^{4}b^{8}\mu^{3}-2094893830R^{6}a^{6}b^{6}-648770080R^{4}a^{4}b^{8}\mu^{2}-1897455R^{2}a^{2}b^{10}\mu^{4}+1692606790R^{4}a^{4}b^{8}\mu +15901200R^{2}a^{2}b^{10}\mu^{3}\\ & -1185959530R^{4}a^{4}b^{8}-170432850R^{2}a^{2}b^{10}\mu^{2}+300751920R^{2}a^{2}b^{10}\mu +2910600b^{12}\mu^{3}-144322815R^{2}a^{2}b^{10}-8731800b^{12}\mu^{2}+8731800b^{12}\mu -2910600b^{12}] \end{array}$$

$$\begin{array}{ll} B_{1} & =-4a^{2}b^{2}C_{0}[M2]\;[-51233212800R^{10}a^{12}b^{4}\mu^{4}C_{0}+84761994240R^{10}a^{12}b^{4}\mu^{3}C_{0}+6662401920R^{8}a^{10}b^{6}\mu^{5}C_{0}-18965802240R^{10}a^{12}b^{4}\mu^{2}C_{0}-32218974720R^{8}a^{10}b^{6}\mu^{4}C_{0}\\ & -82867200R^{6}a^{8}b^{8}\mu^{6}C_{0}-11421527040R^{10}a^{12}b^{4}\mu C_{0}+1796473574400R^{8}a^{10}b^{6}\mu^{3}C_{0}+757927680R^{6}a^{8}b^{8}\mu^{5}C_{0}-3141452160R^{10}a^{12}b^{4}C_{0}\\ & -1543860046080R^{8}a^{10}b^{6}\mu^{2}C_{0}-438345448576R^{6}a^{8}b^{8}\mu^{4}C_{0}+22667164800R^{8}a^{10}b^{6}\mu C_{0}+619593390848R^{6}a^{8}b^{8}\mu^{3}C_{0}+6732289920R^{4}a^{6}b^{10}\mu^{5}C_{0}\\ & +3924320400R^{11}a^{10}\mu^{4}-249724120320R^{8}a^{10}b^{6}C_{0}-3363679726592R^{6}a^{8}b^{8}\mu^{2}C_{0}-29057364480R^{4}a^{6}b^{10}\mu^{4}C_{0}-11772961200R^{11}a^{10}\mu^{3}\\ & -2747519775R^{9}a^{8}b^{2}\mu^{5}+6499828303360R^{6}a^{8}b^{8}\mu C_{0}+1167536967680R^{4}a^{6}b^{10}\mu^{3}C_{0}+11772961200R^{11}a^{10}\mu^{2}+5910264360R^{9}a^{8}b^{2}\mu^{4}\\ & +39167700R^{7}a^{6}b^{4}\mu^{6}-3839509173120R^{6}a^{8}b^{8}C_{0}-1533653195520R^{4}a^{6}b^{10}\mu^{2}C_{0}-25592985600R^{2}a^{4}b^{12}\mu^{4}C_{0}-3924320400R^{11}a^{10}\mu \\ & -170885324610R^{9}a^{8}b^{2}\mu^{3}-230542026R^{7}a^{6}b^{4}\mu^{5}+3710291080320R^{4}a^{6}b^{10}\mu C_{0}+55871854080R^{2}a^{4}b^{12}\mu^{3}C_{0}+362500178040R^{9}a^{8}b^{2}\mu^{2}\\ & +179449795239R^{7}a^{6}b^{4}\mu^{4}-4122350155520R^{4}a^{6}b^{10}C_{0}-534334993920R^{2}a^{4}b^{12}\mu^{2}C_{0}-222247840815R^{9}a^{8}b^{2}\mu -94065997740R^{7}a^{6}b^{4}\mu^{3}\\ & -2908201725R^{5}a^{4}b^{6}\mu^{5}+1104016704000R^{2}a^{4}b^{12}\mu C_{0}+10090080000a^{2}b^{14}\mu^{3}C_{0}+27470242800R^{9}a^{8}b^{2}+264483235302R^{7}a^{6}b^{4}\mu^{2}\\ & +10320953214R^{5}a^{4}b^{6}\mu^{4}-599960578560R^{2}a^{4}b^{12}C_{0}-32691859200a^{2}b^{14}\mu^{2}C_{0}-1610465163450R^{7}a^{6}b^{4}\mu -587435192058R^{5}a^{4}b^{6}\mu^{3}\\ & +35113478400a^{2}b^{14}\mu C_{0}+1260789504975R^{7}a^{6}b^{4}+297067958616R^{5}a^{4}b^{6}\mu^{2}+12342284955R^{3}a^{2}b^{8}\mu^{4}-12511699200a^{2}b^{14}C_{0}\\ & -908454987297R^{5}a^{4}b^{6}\mu -22016824830R^{3}a^{2}b^{8}\mu^{3}+1388061524850R^{5}a^{4}b^{6}+218430051840R^{3}a^{2}b^{8}\mu^{2}-418085798130R^{3}a^{2}b^{8}\mu \\ & -4741887150Rb^{10}\mu^{3}+209330286165R^{3}a^{2}b^{8}+13898634750Rb^{10}\mu^{2}-13571608050Rb^{10}\mu \\ & +4414860450Rb^{10}]/14157[2910600R^{12}a^{12}\mu^{3}-8731800R^{12}a^{12}\mu^{2}-1897455R^{10}a^{10}b^{2}\mu^{4}+8731800R^{12}a^{12}\mu +15901200R^{10}a^{10}b^{2}\mu^{3}\\ & +29050R^{8}a^{8}b^{4}\mu^{5}-2910600R^{12}a^{12}-170432850R^{10}a^{10}b^{2}\mu^{2}-1480150R^{8}a^{8}b^{4}\mu^{4}+300751920R^{10}a^{10}b^{2}\mu +143573920R^{8}a^{8}b^{4}\mu^{3}\\ & +16600R^{6}a^{6}b^{6}\mu^{5}-144322815R^{10}a^{10}b^{2}-648770080R^{8}a^{8}b^{4}\mu^{2}-4259394R^{6}a^{6}b^{6}\mu^{4}+1692606790R^{8}a^{8}b^{4}\mu +102263512R^{6}a^{6}b^{6}\mu^{3}\\ & +29050R^{4}a^{4}b^{8}\mu^{5}-1185959530R^{8}a^{8}b^{4}-1532919728R^{6}a^{6}b^{6}\mu^{2}-1480150R^{4}a^{4}b^{8}\mu^{4}+3190654040R^{6}a^{6}b^{6}\mu +143573920R^{4}a^{4}b^{8}\mu^{3}\\ & -2094893830R^{6}a^{6}b^{6}-648770080R^{4}a^{4}b^{8}\mu^{2}-1897455R^{2}a^{2}b^{10}\mu^{4}+1692606790R^{4}a^{4}b^{8}\mu +15901200R^{2}a^{2}b^{10}\mu^{3}-1185959530R^{4}a^{4}b^{8}\\ & -170432850R^{2}a^{2}b^{10}\mu^{2}+300751920R^{2}a^{2}b^{10}\mu +2910600b^{12}\mu^{3}-144322815R^{2}a^{2}b^{10}-8731800b^{12}\mu^{2}+8731800b^{12}\mu -2910600b^{12}] \end{array}$$

$$\begin{array}{ll} B_{2} & =-8a^{6}C_{0}[110073600R^{12}a^{12}b^{2}\mu^{3}C_{0}-330220800R^{12}a^{12}b^{2}\mu^{2}C_{0}-135703680R^{10}a^{10}b^{4}\mu^{4}C_{0}+330220800R^{12}a^{12}b^{2}\mu C_{0}+795782400R^{10}a^{10}b^{4}\mu^{3}C_{0}\\ & +52438400R^{8}a^{8}b^{6}\mu^{5}C_{0}-110073600R^{12}a^{12}b^{2}C_{0}-6641402880R^{10}a^{10}b^{4}\mu^{2}C_{0}-372031744R^{8}a^{8}b^{6}\mu^{4}C_{0}+11438273280R^{10}a^{10}b^{4}\mu C_{0}\\ & +9103828608R^{8}a^{8}b^{6}\mu^{3}C_{0}+6841856R^{6}a^{6}b^{8}\mu^{5}C_{0}-5456949120R^{10}a^{10}b^{4}C_{0}-31255516544R^{8}a^{8}b^{6}\mu^{2}C_{0}-3448479488R^{6}a^{6}b^{8}\mu^{4}C_{0}\\ & +67284474880R^{8}a^{8}b^{6}\mu C_{0}+16246501888R^{6}a^{6}b^{8}\mu^{3}C_{0}+39459200R^{4}a^{4}b^{10}\mu^{5}C_{0}+26756730R^{11}a^{8}\mu^{4}-44813193600R^{8}a^{8}b^{6}C_{0}\\ & -93467685376R^{6}a^{6}b^{8}\mu^{2}C_{0}-554781184R^{4}a^{4}b^{10}\mu^{4}C_{0}-80270190R^{11}a^{8}\mu^{3}-16945929R^{9}a^{6}b^{2}\mu^{5}+148592168960R^{6}a^{6}b^{8}\mu C_{0}\\ & +33228491392R^{4}a^{4}b^{10}\mu^{3}C_{0}+80270190R^{11}a^{8}\mu^{2}+130810680R^{9}a^{6}b^{2}\mu^{4}-79542081280R^{6}a^{6}b^{8}C_{0}-88011920768R^{4}a^{4}b^{10}\mu^{2}C_{0}\\ & -1717330560R^{2}a^{2}b^{12}\mu^{4}C_{0}-26756730R^{11}a^{8}\mu -1530088560R^{9}a^{6}b^{2}\mu^{3}+1245816R^{7}a^{4}b^{4}\mu^{5}+109728729600R^{4}a^{4}b^{10}\mu C_{0}\\ & +5338924800R^{2}a^{2}b^{12}\mu^{3}C_{0}+2742465726R^{9}a^{6}b^{2}\mu^{2}+1213674891R^{7}a^{4}b^{4}\mu^{4}-48058665600R^{4}a^{4}b^{10}C_{0}-30261623040R^{2}a^{2}b^{12}\mu^{2}C_{0}\\ & -1333178847R^{9}a^{6}b^{2}\mu -5167125678R^{7}a^{4}b^{4}\mu^{3}-10811229R^{5}a^{2}b^{6}\mu^{5}+35100975360R^{2}a^{2}b^{12}\mu C_{0}+1170892800b^{14}\mu^{3}C_{0}\\ & +6936930R^{9}a^{6}b^{2}+14529187530R^{7}a^{4}b^{4}\mu^{2}+31183152R^{5}a^{2}b^{6}\mu^{4}-8460946560R^{2}a^{2}b^{12}C_{0}-2575964160b^{14}\mu^{2}C_{0}\\ & -10882835106R^{7}a^{4}b^{4}\mu -11060080746R^{5}a^{2}b^{6}\mu^{3}+1639249920b^{14}\mu C_{0}+305852547R^{7}a^{4}b^{4}+24034791924R^{5}a^{2}b^{6}\mu^{2}+519038091R^{3}b^{8}\mu^{4}\\ & -234178560b^{14}C_{0}-17752099365R^{5}a^{2}b^{6}\mu +221443794R^{3}b^{8}\mu^{3}+1341404064R^{5}a^{2}b^{6}+7092741942R^{3}b^{8}\mu^{2}\\ & -8854948674R^{3}b^{8}\mu +1021724847R^{3}b^{8}]/429[2910600R^{12}a^{12}\mu^{3}-8731800R^{12}a^{12}\mu^{2}-1897455R^{10}a^{10}b^{2}\mu^{4}+8731800R^{12}a^{12}\mu \\ & +15901200R^{10}a^{10}b^{2}\mu^{3}+29050R^{8}a^{8}b^{4}\mu^{5}-2910600R^{12}a^{12}-170432850R^{10}a^{10}b^{2}\mu^{2}-1480150R^{8}a^{8}b^{4}\mu^{4}+300751920R^{10}a^{10}b^{2}\mu \\ & +143573920R^{8}a^{8}b^{4}\mu^{3}+16600R^{6}a^{6}b^{6}\mu^{5}-144322815R^{10}a^{10}b^{2}-648770080R^{8}a^{8}b^{4}\mu^{2}-4259394R^{6}a^{6}b^{6}\mu^{4}+1692606790R^{8}a^{8}b^{4}\mu \\ & +102263512R^{6}a^{6}b^{6}\mu^{3}+29050R^{4}a^{4}b^{8}\mu^{5}-1185959530R^{8}a^{8}b^{4}-1532919728R^{6}a^{6}b^{6}\mu^{2}-1480150R^{4}a^{4}b^{8}\mu^{4}+3190654040R^{6}a^{6}b^{6}\mu \\ & +143573920R^{4}a^{4}b^{8}\mu^{3}-2094893830R^{6}a^{6}b^{6}-648770080R^{4}a^{4}b^{8}\mu^{2}-1897455R^{2}a^{2}b^{10}\mu^{4}+1692606790R^{4}a^{4}b^{8}\mu +15901200R^{2}a^{2}b^{10}\mu^{3}\\ & -1185959530R^{4}a^{4}b^{8}-170432850R^{2}a^{2}b^{10}\mu^{2}+300751920R^{2}a^{2}b^{10}\mu +2910600b^{12}\mu^{3}-144322815R^{2}a^{2}b^{10}-8731800b^{12}\mu^{2}+8731800b^{12}\mu -2910600b^{12}] \end{array}$$

## Appendix B

*H*_1_ to *H*_5_ are listed as follows, in which, for brevity, let *μ*^−^ be *μ*.

$$\begin{array}{ll} H_{1}\left(\lambda ,\mu \right) & =\frac{199176828100579479150\mu^{2}}{\lambda^{2}}-\frac{351474574712323542480\mu }{\lambda^{2}}+\frac{2217466106819108145\mu^{4}}{\lambda^{2}}-\frac{18582981972037282800\mu^{3}}{\lambda^{2}}\\ & +\frac{168663262476962237985}{\lambda^{2}}-\frac{1978069671742880726010\mu }{\lambda^{4}}+\frac{758186973350261975520\mu^{2}}{\lambda^{4}}-\frac{167788064235071760480\mu^{3}}{\lambda^{4}}\\ & +\frac{1729781448312767850\mu^{4}}{\lambda^{4}}-\frac{33949363965466950\mu^{5}}{\lambda^{4}}+\frac{1385974930543343210070}{\lambda^{4}}-\frac{3728766791457746798760\mu }{\lambda^{6}}\\ & +\frac{1791450938923118705232\mu^{2}}{\lambda^{6}}-\frac{119510540078309708328\mu^{3}}{\lambda^{6}}+\frac{4977752742799522686\mu^{4}}{\lambda^{6}}-\frac{19399636551695400\mu^{5}}{\lambda^{6}}\\ & +\frac{2448203549180070451770}{\lambda^{6}}-\frac{1978069671742880726010\mu }{\lambda^{8}}+\frac{758186973350261975520\mu^{2}}{\lambda^{8}}-\frac{167788064235071760480\mu^{3}}{\lambda^{8}}\\ & +\frac{1729781448312767850\mu^{4}}{\lambda^{8}}-\frac{33949363965466950\mu^{5}}{\lambda^{8}}+\frac{1385974930543343210070}{\lambda^{8}}-\frac{351474574712323542480\mu }{\lambda^{10}}\\ & +\frac{199176828100579479150\mu^{2}}{\lambda^{10}}-\frac{18582981972037282800\mu^{3}}{\lambda^{10}}+\frac{2217466106819108145\mu^{4}}{\lambda^{10}}+\frac{168663262476962237985}{\lambda^{10}}\\ & -\frac{10204442556752644200\mu }{\lambda^{12}}+\frac{10204442556752644200\mu^{2}}{\lambda^{12}}-\frac{3401480852250881400\mu^{3}}{\lambda^{12}}+\frac{3401480852250881400}{\lambda^{12}}\\ & +10204442556752644200\mu^{2}-3401480852250881400\mu^{3}-10204442556752644200\mu +3401480852250881400 \end{array}$$

$$\begin{array}{ll} H_{2}\left(\lambda ,\mu \right) & =\frac{80455987092344992128\mu^{2}}{\lambda^{2}}-\frac{193485524003130078720\mu }{\lambda^{2}}-\frac{31498764224966438400\mu^{4}}{\lambda^{2}}+\frac{48825171651306226176\mu^{3}}{\lambda^{2}}\\ & +\frac{93207509358313109760}{\lambda^{2}}+\frac{2880628992405964800\mu^{5}}{\lambda^{2}}-\frac{385008866273775744\mu^{6}}{\lambda^{2}}-\frac{889677341822370157056\mu }{\lambda^{4}}\\ & +\frac{175264953575370227712\mu^{2}}{\lambda^{4}}+\frac{187702091976537515520\mu^{3}}{\lambda^{4}}-\frac{109841996356766099712\mu^{4}}{\lambda^{4}}+\frac{26558705402032167936\mu^{5}}{\lambda^{4}}\\ & +\frac{610052314515833629440}{\lambda^{4}}-\frac{64358024148887040\mu^{6}}{\lambda^{4}}+\frac{5630733511603200\mu^{7}}{\lambda^{4}}-\frac{421710394343382144\mu^{6}}{\lambda^{6}}\\ & -\frac{1522583254870884670464\mu }{\lambda^{6}}+\frac{382682695949458160256\mu^{2}}{\lambda^{6}}+\frac{436162518546143380992\mu^{3}}{\lambda^{6}}-\frac{203696893485704691072\mu^{4}}{\lambda^{6}}\\ & +\frac{3130040637298358784\mu^{5}}{\lambda^{6}}+\frac{1018613548263068005248}{\lambda^{6}}-\frac{773969744755438133760\mu }{\lambda^{8}}+\frac{128360929274195904000\mu^{2}}{\lambda^{8}}\\ & +\frac{94946445493607904768\mu^{3}}{\lambda^{8}}-\frac{17153932050179248896\mu^{4}}{\lambda^{8}}+\frac{3612385551541358592\mu^{5}}{\lambda^{8}}+\frac{564203916486272215296}{\lambda^{8}}\\ & -\frac{142133177391572150784\mu }{\lambda^{10}}+\frac{75849755706354389760\mu^{2}}{\lambda^{10}}+\frac{353454032154263040\mu^{3}}{\lambda^{10}}-\frac{2656674029399854464\mu^{4}}{\lambda^{10}}\\ & +\frac{68586641682463352448}{\lambda^{10}}-\frac{4149589413424815360\mu }{\lambda^{12}}+\frac{4149589413424815360\mu^{2}}{\lambda^{12}}-\frac{1383196471141605120\mu^{3}}{\lambda^{12}}\\ & +\frac{1383196471141605120}{\lambda^{12}}+5332308027496369920\mu^{2}-200477857070050560\mu^{3}-5923667334532147200\mu \\ & +591359307035777280\mu^{5}-1774077921107331840\mu^{4}+1974555778177382400 \end{array}$$

$$\begin{array}{ll} H_{3}\left(\lambda ,\mu \right) & =\frac{1}{1-\mu^{2}}[124397014025175091200\mu^{2}\lambda^{4}-186595521037762636800\mu \lambda^{4}+62198507012587545600\lambda^{4}+62198507012587545600\mu^{5}\lambda^{4}\\ & -186595521037762636800\mu^{4}\lambda^{4}+629046350846243890560\mu^{2}\lambda^{2}-6533589663904066283520\mu \lambda^{2}-3708168631336054005120\mu^{4}\lambda^{2}\\ & +6158244560979620229120\mu^{3}\lambda^{2}+3119670232157359520640\lambda^{2}+375345102924446054400\mu^{5}\lambda^{2}-40547951667549406080\mu^{6}\lambda^{2}\\ & -\frac{18010971797435210385792\mu^{2}}{\lambda^{2}}-\frac{95278937469810978723840\mu }{\lambda^{2}}-\frac{44172686641179046414464\mu^{4}}{\lambda^{2}}+\frac{91000584721944292082688\mu^{3}}{\lambda^{2}}\\ & +\frac{62333302605773326807680}{\lambda^{2}}+\frac{4277643275444224637952\mu^{5}}{\lambda^{2}}-\frac{149644167159070007424\mu^{6}}{\lambda^{2}}+\frac{709472422462003200\mu^{7}}{\lambda^{2}}\\ & -\frac{111302641816114461672960\mu }{\lambda^{4}}-\frac{29821497055950221766144\mu^{2}}{\lambda^{4}}+\frac{103917629415515621561856\mu^{3}}{\lambda^{4}}-\frac{46331542024747273502208\mu^{4}}{\lambda^{4}}\\ & +\frac{7383568117453113890304\mu^{5}}{\lambda^{4}}+\frac{76268312096201369464320}{\lambda^{4}}-\frac{115273015503874195968\mu^{6}}{\lambda^{4}}+\frac{1444283145726220800\mu^{7}}{\lambda^{4}}\\ & -\frac{149644167159070007424\mu^{6}}{\lambda^{6}}-\frac{95278937469810978723840\mu }{\lambda^{6}}-\frac{18010971797435210385792\mu^{2}}{\lambda^{6}}+\frac{91000584721944292082688\mu^{3}}{\lambda^{6}}\\ & -\frac{44172686641179046414464\mu^{4}}{\lambda^{6}}+\frac{4277643275444224637952\mu^{5}}{\lambda^{6}}+\frac{62333302605773326807680}{\lambda^{6}}+\frac{709472422462003200\mu^{7}}{\lambda^{6}}\\ & -\frac{54800547436318844160\mu^{6}}{\lambda^{8}}+\frac{620788369654252800\mu^{7}}{\lambda^{8}}-\frac{40029563033983288846080\mu }{\lambda^{8}}-\frac{11036318578744391412480\mu^{2}}{\lambda^{8}}\\ & +\frac{36705066724433815868160\mu^{3}}{\lambda^{8}}-\frac{16076979955424534065920\mu^{4}}{\lambda^{8}}+\frac{3323875521179818725120\mu^{5}}{\lambda^{8}}+\frac{27168099081605244322560}{\lambda^{8}}\\ & +\frac{375345102924446054400\mu^{5}}{\lambda^{10}}-\frac{40547951667549406080\mu^{6}}{\lambda^{10}}-\frac{6533589663904066283520\mu }{\lambda^{10}}+\frac{629046350846243890560\mu^{2}}{\lambda^{10}}\\ & +\frac{6158244560979620229120\mu^{3}}{\lambda^{10}}-\frac{3708168631336054005120\mu^{4}}{\lambda^{10}}+\frac{3119670232157359520640}{\lambda^{10}}+\frac{62198507012587545600\mu^{5}}{\lambda^{12}}\\ & -\frac{186595521037762636800\mu }{\lambda^{12}}-\frac{186595521037762636800\mu^{4}}{\lambda^{12}}+\frac{124397014025175091200\mu^{2}}{\lambda^{12}}+\frac{124397014025175091200\mu^{3}}{\lambda^{12}}\\ & +\frac{62198507012587545600}{\lambda^{12}}-11036318578744391412480\mu^{2}+36705066724433815868160\mu^{3}-40029563033983288846080\mu \\ & +3323875521179818725120\mu^{5}-16076979955424534065920\mu^{4}+27168099081605244322560-54800547436318844160\mu^{6}\\ & +620788369654252800\mu^{7}+124397014025175091200\mu^{3}\lambda^{4}] \end{array}$$

$$\begin{array}{ll} H_{4}\left(\lambda ,\mu \right) & =11852714441414246400\mu^{2}\lambda^{2}-3950904813804748800\mu \lambda^{2}+3950904813804748800\mu^{4}\lambda^{2}-11852714441414246400\mu^{3}\lambda^{2}\\ & +\frac{1431097447076741283840\mu^{2}}{\lambda^{2}}-\frac{417519642156446269440\mu }{\lambda^{2}}+\frac{367848461802280402944\mu^{4}}{\lambda^{2}}-\frac{1037941168689618665472\mu^{3}}{\lambda^{2}}\\ & -\frac{329617315193148702720}{\lambda^{2}}-\frac{16398140832033472512\mu^{5}}{\lambda^{2}}+\frac{2530357992225423360\mu^{6}}{\lambda^{2}}+\frac{937332920140188549120\mu }{\lambda^{4}}\\ & +\frac{2335983705328390520832\mu^{2}}{\lambda^{4}}-\frac{2154407358883037626368\mu^{3}}{\lambda^{4}}+\frac{621680913047229235200\mu^{4}}{\lambda^{4}}-\frac{169383928325750341632\mu^{5}}{\lambda^{4}}\\ & -\frac{1862447003416382423040}{\lambda^{4}}+\frac{365181605764005888\mu^{6}}{\lambda^{4}}-\frac{35738870552985600\mu^{7}}{\lambda^{4}}+\frac{2646832263124008960\mu^{6}}{\lambda^{6}}\\ & +\frac{3429746730417442406400\mu }{\lambda^{6}}+\frac{639461530992380608512\mu^{2}}{\lambda^{6}}-\frac{2316284756409671663616\mu^{3}}{\lambda^{6}}+\frac{940566332992044515328\mu^{4}}{\lambda^{6}}\\ & -\frac{17879177442769403904\mu^{5}}{\lambda^{6}}-\frac{2969168802186701537280}{\lambda^{6}}+\frac{2041193114473393520640\mu }{\lambda^{8}}+\frac{62692180505155289088\mu^{2}}{\lambda^{8}}\\ & -\frac{551849050795970543616\mu^{3}}{\lambda^{8}}+\frac{77958068457829367808\mu^{4}}{\lambda^{8}}-\frac{17485111999324569600\mu^{5}}{\lambda^{8}}-\frac{1612509200641083064320}{\lambda^{8}}\\ & +\frac{402378274688877527040\mu }{\lambda^{10}}-\frac{204577015833728778240\mu^{2}}{\lambda^{10}}-\frac{14367597320518041600\mu^{3}}{\lambda^{10}}+\frac{12469637235033538560\mu^{4}}{\lambda^{10}}\\ & -\frac{195903298769664245760}{\lambda^{10}}+\frac{11852714441414246400\mu }{\lambda^{12}}-\frac{11852714441414246400\mu^{2}}{\lambda^{12}}+\frac{3950904813804748800\mu^{3}}{\lambda^{12}}\\ & -\frac{3950904813804748800}{\lambda^{12}}+364736127106760048640\mu^{2}-218794917301175255040\mu^{3}-162864323511159029760\mu \\ & -6351436556025200640\mu^{5}+30383500995322675200\mu^{4}-7108950733723238400 \end{array}$$

$$\begin{array}{ll} H_{5}\left(\lambda ,\mu \right) & =8963671686591283200\mu^{2}\lambda^{4}-2987890562197094400\mu^{3}\lambda^{4}-8963671686591283200\mu \lambda^{4}+2987890562197094400\lambda^{4}\\ & +149277015891907706880\mu^{2}\lambda^{2}-303807001461931376640\mu \lambda^{2}-12715560541964206080\mu^{4}\lambda^{2}+18705070615324262400\mu^{3}\lambda^{2}\\ & +148540475496663613440\lambda^{2}-\frac{813612111564092997632\mu^{2}}{\lambda^{2}}-\frac{2969300415537962024960\mu }{\lambda^{2}}-\frac{946220140857133105152\mu^{4}}{\lambda^{2}}\\ & +\frac{2288127548216441831424\mu^{3}}{\lambda^{2}}+\frac{2623361571335490764800}{\lambda^{2}}+\frac{25412852208170434560\mu^{5}}{\lambda^{2}}-\frac{3833933856075939840\mu^{6}}{\lambda^{2}}\\ & -\frac{2550473052227475865600\mu }{\lambda^{4}}-\frac{2179018170489689341952\mu^{2}}{\lambda^{4}}+\frac{2687448145649664524288\mu^{3}}{\lambda^{4}}-\frac{895236976724945141760\mu^{4}}{\lambda^{4}}\\ & +\frac{251659568284176482304\mu^{5}}{\lambda^{4}}+\frac{3036603210819006627840}{\lambda^{4}}-\frac{535541690841169920\mu^{6}}{\lambda^{4}}+\frac{50408544691814400\mu^{7}}{\lambda^{4}}\\ & -\frac{3833933856075939840\mu^{6}}{\lambda^{6}}-\frac{2969300415537962024960\mu }{\lambda^{6}}-\frac{813612111564092997632\mu^{2}}{\lambda^{6}}+\frac{2288127548216441831424\mu^{3}}{\lambda^{6}}\\ & -\frac{946220140857133105152\mu^{4}}{\lambda^{6}}+\frac{25412852208170434560\mu^{5}}{\lambda^{6}}+\frac{2623361571335490764800}{\lambda^{6}}-\frac{1534067943304750694400\mu }{\lambda^{8}}\\ & -\frac{174405462194021990400\mu^{2}}{\lambda^{8}}+\frac{526768376344026808320\mu^{3}}{\lambda^{8}}-\frac{82013551836203581440\mu^{4}}{\lambda^{8}}+\frac{19891873079466393600\mu^{5}}{\lambda^{8}}\\ & +\frac{1243826707911483064320}{\lambda^{8}}-\frac{303807001461931376640\mu }{\lambda^{10}}+\frac{149277015891907706880\mu^{2}}{\lambda^{10}}+\frac{18705070615324262400\mu^{3}}{\lambda^{10}}\\ & -\frac{12715560541964206080\mu^{4}}{\lambda^{10}}+\frac{148540475496663613440}{\lambda^{10}}-\frac{8963671686591283200\mu }{\lambda^{12}}+\frac{8963671686591283200\mu^{2}}{\lambda^{12}}\\ & -\frac{2987890562197094400\mu^{3}}{\lambda^{12}}+\frac{2987890562197094400}{\lambda^{12}}-174405462194021990400\mu^{2}+526768376344026808320\mu^{3}\\ & -1534067943304750694400\mu +19891873079466393600\mu^{5}-82013551836203581440\mu^{4}+1243826707911483064320 \end{array}$$
